# Overexpression of *BvKUP13* from sugar beet increased salt tolerance in transgenic *Arabidopsis thaliana*

**DOI:** 10.3389/fpls.2026.1736699

**Published:** 2026-02-23

**Authors:** Shuai Wang, Yuzhu Cui, Jiamin Cheng, Yuxin Wang, Yaqing Sun, Guolong Li, Shaoying Zhang, Ningning Li

**Affiliations:** College of Agriculture, Inner Mongolia Agricultural University, Hohhot, China

**Keywords:** BvKUP13, functional identification, salt stress, sugar beet, transgenic *Arabidopsis*

## Abstract

**Introduction:**

The KUP/HAK/KT family is the largest group of potassium ion transporters in plants and plays a central role in K^+^ uptake, transport, and abiotic stress responses. However, the function of individual KUP genes in salt-tolerant crops remains under explored. In this study, *BvKUP13* was isolated from the salt-tolerant beet variety AK3018.

**Methods:**

We cloned and analyzed the sequence, subcellular localization, and expression profile of *BvKUP13* under salt stress. Transgenic *Arabidopsis thaliana* plants overexpressing *BvKUP13* were generated to evaluate its role in salt stress tolerance.

**Results:**

*BvKUP13* encodes a protein of 732 amino acids, shows striking sequence similarity to KUP proteins from *Chenopodium quinoa* and *Spinacia oleracea*, and is localized to the endoplasmic reticulum membrane. The gene is predominantly expressed in leaves and is upregulated by NaCl treatment. Overexpression in Arabidopsis significantly improved salt tolerance, as indicated by enhanced photosynthetic efficiency, maintained Na^+^/K^+^ balance, elevated antioxidant enzyme activity, and higher osmolyte levels. Reduced MDA and ROS accumulation further supported the protective role of *BvKUP13* under salt stress.

**Discussion:**

These findings demonstrate that *BvKUP13* enhances salt tolerance by regulating ion homeostasis and strengthening physiological and biochemical stress responses. *BvKUP13* is a potential candidate gene for engineering salt-tolerant sugar beet varieties.

## 1 Introduction

Soil salinization is a major factor that limits global soil productivity, food security, and ecosystems (Phankamolsil et al., 2024). Among strategies to improve the productivity of salinized soils, enhancing plants’ inherent resistance is crucial (Daba, 2025). Salt stress increases the concentration of toxic ions in plants, leading to ion toxicity. This, in turn, affects the root system’s ability to absorb essential minerals, disrupts physiological and metabolic activities, inhibits growth, and may even cause premature senescence or death (Tahjib-Ul-Arif et al., 2023). In response to salt stress, plants produce signaling molecules that activate multiple related pathways, including osmotic regulation, ion transport and compartmentalization, photosynthesis regulation, ROS scavenging, and protein ubiquitination, enabling the plant’s adaptive response to salt stress (Behera and Hembram, 2021; Ibrahimova et al., 2021). Potassium is an essential nutrient that participates in various metabolic pathways (Sivakumar et al., 2022). It plays a key role in maintaining cell turgor pressure, promoting cell elongation, and regulating osmotic balance, among other physiological functions (Anschütz et al., 2014). The absorption and transport of K^+^ in plants are mediated primarily by potassium transporters and channels. Potassium transporters, classified into three major families (KUP/HAK/KT, HKT, and CPA), are most abundant in plants, with the KUP/HAK/KT family being the largest, and its members involved in diverse roles in potassium uptake, transport, salt tolerance, osmotic regulation, as well as controlling root morphology and shoot phenotype (Grabov, 2007; Li et al., 2018).

The KT/HAK/KUP family is known to be an important group of high-affinity K^+^ transporters in many species. The expression of these genes shows significant tissue specificity and is influenced by various factors. They are involved in K^+^ uptake and transport, as well as in regulating the Na^+^/K^+^ ratio to improve salt tolerance (Liu K. et al., 2025; Wang J. et al., 2024). For example, under salt stress, overexpression of the *ZmHAK17* in maize promoted Na^+^ efflux from the embryonic root, reduced its accumulation in the embryo, and enhanced seed germination and seedling growth, compared to both WT and *zmhak17* plants (Wang L. et al., 2024). Overexpression of the *PbKT8* from pear promoted *Arabidopsis thaliana* growth under normal potassium conditions, regulated K^+^ uptake under potassium deficiency, and accelerated early flowering (Shi et al., 2019). The *atkup9* mutant showed decreased chlorophyll content in seedlings, yellowing of rosette leaves, and altered K^+^ distribution in leaves and uptake in roots under low-K^+^ conditions (Yamanashi et al., 2022). *MiHAK14* was highly expressed in mango roots, and its overexpression in *Arabidopsis* enhanced tolerance to K^+^ depletion and NaCl stress, improved K^+^ nutrition, and boosted reactive oxygen species (ROS) scavenging ability (Zhang Y. et al., 2022). *PtKUP10* overexpression in *Arabidopsis* enhanced salt tolerance by increasing potassium accumulation in the stem and decreasing sodium content in both stem and roots (Jin et al., 2024). *TaHAK1* promoted seedling growth by sequestering Cs in the root cell wall and modulating Cs distribution. Under Cs-contaminated soil conditions, *TaHAK1* enhanced *Arabidopsis* tolerance to Cs^+^ by reducing Cs accumulation in tissues and increasing K^+^ content (Liu et al., 2024). Plants expressing *AtHAK5* in the *athak5akt1* double mutant accumulated less Na and Cs^+^, leading to a lower Na^+^/Cs^+^ ratio and a reduced Cs^+^/K^+^ ratio (Jiménez-Estévez et al., 2024). Low potassium induced *SiHAK1* K^+^ uptake activity in CY162 yeast cells and *Arabidopsis athak5* mutants. In a low-K^+^, high-Na^+^ environment, *SiHAK1*’s transport activity was regulated by both external K^+^ supply and internal K^+^ content (Zhang et al., 2018).

Sugar beet (*Beta vulgaris* L.), the second largest sugar crop, is widely cultivated in the arid and semi-arid regions of northern China. It has strong salt tolerance and drought resistance, making it an ideal candidate for exploring salt-tolerant genes (Alavilli et al., 2023). Studies have shown that during its vegetative growth phase, beet absorbed large amounts of Na^+^ from the soil, transporting most of it to the aboveground leaves, where it replaced K^+^ for osmotic regulation in the vacuole, thus adapting to saline environments (Wakeel et al., 2009). The primary goals of beet cultivation are high yield, high sugar content, and strong stress resistance. However, beet salt tolerance has limitations. Therefore, identifying and verifying key genes involved in the salt stress response, along with understanding the physiological and molecular regulatory mechanisms, is crucial for developing salt-tolerant beet varieties and mitigating the impact of soil salinization on beet growth and yield (Ribeiro et al., 2024; Wang Y. et al., 2024).

In our previous study, 50 beet varieties were screened to identify the salt-tolerant line AK3018 and the salt-sensitive line IM1162. Their salt tolerance mechanisms were elucidated through integrated physiological and transcriptomic analyses. Transcriptomic analysis revealed significant enrichment of salt stress-responsive genes in pathways associated with ion transmembrane transport, biosynthesis of osmolytes, antioxidant enzyme activity, and chlorophyll biosynthesis. Accordingly, the salt-tolerant variety AK3018maintained better ion homeostasis under salt stress,as evidenced by a higher leaf K^+^/Na^+^ ratio, greater accumulation of osmolytes, and elevated antioxidant enzyme activities (Li et al., 2025). In this work, we systematically investigated the role of *BvKUP13* in salt tolerance by constructing an overexpression system in *Arabidopsis*. The aim was to reveal its synergistic role in maintaining ion homeostasis, alleviating oxidative damage, and regulating osmotic balance, thus offering new genetic resources and theoretical support for improving salt tolerance in crops.

## 2 Materials and methods

### 2.1 Plant materials and treatments

The beetroot (*Beta vulgaris L. ssp. vulgaris*) seeds used in this study were of the commercial cultivar ‘AK3018’. The seeds were purchased from Jinyangpu Agricultural Co., LTD, located in Beijing, China. The seeds were obtained as a commercial product for agricultural use and did not require any specific permission for collection. Formal identification of the plant material was conducted by the seed supplier based on the cultivar characteristics described in the product catalog. As the material is a widely available commercial cultivar, no voucher specimen was deposited. This study complied with relevant institutional, national, and international guidelines and legislation for the use of cultivated plant material in experimental research.

Seeds were sown in seed trays and placed in a growth chamber with a light/dark cycle of 16 hours at 26 °C and 8 hours at 22 °C. Seedlings with 6–8 leaves and consistent growth vigor were selected for NaCl root irrigation treatment at concentrations of 0, 200, 400, 600, and 800 mM. After 7 days, roots and leaves were harvested, immediately frozen in liquid nitrogen, and stored at −80 °C for later use. *Arabidopsis* seeds of the Columbia WT (Col-0) were used. Prior to sowing, the seeds were disinfected and then sown on MS medium. The 15-day-old seedlings were transferred to seedling trays and placed in a growth chamber for continued cultivation.

### 2.2 Cloning and sequence analysis of *BvKUP13*

Total RNA was extracted from salt-tolerant beet leaves using the TransZol Plant reagent kit (Kangwei Century) and then reverse transcribed to synthesize complementary DNA (cDNA). Specific primers *BvKUP13*-F/R, designed by NCBI (accession number: XM010671674.3), were used for PCR amplification, with the cDNA sequence from salt-tolerant beet leaves serving as the template. The target band was obtained, purified using gel electrophoresis, extracted and ligated into the T vector pEASY-T1. Following identification through colony PCR, the construct was submitted to Beijing Huada Biotechnology Co., Ltd. for sequencing, and the correctly sequenced bacterial cultures were preserved. The conserved domains of the BvKUP13 protein were analyzed using NCBI’s Conserved Domain Database (CDD). The predicted secondary structure of the protein was analyzed using SWISS-MODEL, and its transmembrane domains were assessed using TMHMM. The sequence alignment and conservation of *BvKUP13* and homologs were visualizedusing Espript. The accession numbers of KUP homologs from other species were: *Chenopodium quinoa* (CAN99589.XP021774497.1; Cq), *Triticum aestivum* (XP044424600.1; Ta), *Hordeum vulgare* (KAE8804611.1; Hv), *Arabidopsis thaliana* (OAPO8615.1; At), and *Malus domestica* (XP008346085.2; Md). The phylogenetic tree was constructed using the Neighbor-Joining method in MEGA orrelevant software.

### 2.3 Subcellular localization analysis of *BvKUP1*3

Target gene amplification was performedusing gfp*BvKUP13*-R/F primers, and the full-length *BvKUP13* cDNA was amplified viaPCR. The resulting product was ligated into the pAN580 expression vector, which had been pre-digested with *Bam*HI and *Xba*I restriction enzymes, yielding the recombinant construct BvKUP13-pAN580. Nicotiana benthamiana plants were grown under controlled conditions at 25 °C for 15-20 days. Leaf-derived protoplasts were isolated via enzymatic digestion of young leaf tissue using standard protocols. A total of 100 µL of the prepared protoplast suspension was mixed with 10 µL of plasmid DNA and 100 µL of PEG4000 solution (w/v). After incubation, the mixture was washed to remove residual PEG, and the supernatant was discarded, and approximately 100 µL of protoplasts were retained. The samples were then observed using either fluorescence or laser scanning confocal microscopy. Excitation wavelengths of 488 nm and 561 nm were applied to detect GFP and the endoplasmic reticulum marker, respectively.

The full-length *BvKUP13* was amplified by PCR using the *GFPBvKUP13*-R/F primers. The amplified product was recovered from the gel and recombined with the linearized pBWA(V)H2S-ccdB-egfp vector, which had been digested with *Bsa*I/*Eco*31I enzymes, to obtain the recombinant vector pBWA(V)H2S-BvKUP13-GFP. The endoplasmic reticulum (ER) marker plasmid was transformed into Agrobacterium, and then co-suspended with Agrobacterium harboring the target gene. Prior to injection, the two Agrobacterium cultures were mixed at a 1:1 ratio and infiltrated into one-month-old tobacco leaves. Observations were performed after two days of cultivation under low-light conditions.

### 2.4 Real-time PCR analysis

Real-time PCR was used to measure the relative expression levels of *BvKUP13* in the roots, petioles, and leaves of the salt-tolerant beet variety AK3018, as well as in the roots and leaves of seedlings after 7 days of NaCl stress at concentrations of 0, 200, 400, 600, and 800 mM. Changes in the expression levels of *BvKUP13* in the roots and leaves were also measured after 48 hours of treatment with 600 mM NaCl. Specific primers for quantitative real-time PCR were designed based on the cloned *BvKUP13* sequence: q*BvKUP13*-F/R. The beet *Actin* gene served as the internal reference, with primers designed as *BvActin*-F/R. Total RNA was extracted from both aerial and root tissues using the TransZol Plant reagent kit, followed by reverse transcription into cDNA. The cDNA was subsequently diluted 5-fold and used as the template for real-time quantitative PCR. Each treatment was performed with three biological replicates and three technical replicates. Relative gene expression was calculated using the 2^−ΔΔCt^ method.

### 2.5 Screening and identification of transgenic *Arabidopsis*

The full-length *BvKUP13* cDNA was amplified using pBI101-*BvKUP13*-F/R primers, which target the gene. The PCR products were then inserted into the pBI101-km-35S::Gus-Hm vector, previously linearized by *Bam*HI restriction enzymes, to generate the recombinant plasmid. This plasmid was subsequently transformed into the *Agrobacterium tumefaciens* strain GV3101. The Agrobacterium-mediated floral dip method was used to transform WT, generating T_0_ generation seeds. All T_0_ generation seeds were sown on MS solid medium supplemented with Kanamycin (50 mg/L) to generate T1 generation seeds. DNA was extracted from the target lines, and PCR was performed using kanamycin resistance primers *Kan*-F/R to identify and screen positive lines. These positive lines were subsequently self-pollinated to obtain T_3_ generation *Arabidopsis* lines overexpressing *BvKUP13*. RNA was extracted and reverse transcribed, utilizing WT *Arabidopsis* as a control and *AtActin* as the reference gene, to assess the expression of *BvKUP13* in three homozygous transgenic lines. All special primer sequences are shown in Table (S1).

### 2.6 Salt resistance analysis of transgenic *Arabidopsis*

The concentrations of NaCl used in the experiments were determined through preliminary trials to reflect the specific salt sensitivity of each developmental stage under investigation. Homozygous transgenic *Arabidopsis* seeds were sown on MS medium supplemented with 0, 50, or 100 mM NaCl for germination and survival assays. Germination rates were recorded after 7 days, and survival rates after 14 days. For root development analysis, 15-day-old seedlings grown on standard MS medium were transferred to vertical MS plates containing 0, 75, or 150 mM NaCl and cultivated for an additional 7 days, after which root lengths were measured. Following 20 days of plate growth, seedlings were transplanted into soil and grown for another 20 days. Subsequently, these soil-grown plants were irrigated with 0, 100, or 200 mM NaCl solutions for 7 days prior to phenotypic evaluation. Fresh and dry weights were measured using an electronic balance. Leaf discs (1 cm in diameter) were collected for senescence assays under 300 mM NaCl treatment. Chlorophyll content was assessed using a SPAD-502 meter, and the maximum photochemical efficiency of PSII (Fv/Fm) was measured using a chlorophyll fluorescence imaging system. For ion content analysis, plant tissues from the 200 mM NaCl treatment were dried at 80 °C to constant weight, ground into powder, and digested in 10% nitric acid. Na^+^ and K^+^ contents were quantified using a flame photometer. Ion-specific fluorescence probes were obtained from Shanghai Maokang Biotechnology Co., Ltd., and staining was performed according to the manufacturer’s instructions. Fluorescence signals were observed using a stereo fluorescence microscope. Additionally, fresh leaves were stained with NBT, DAB, and Evans blue to assess reactive ROS accumulation and cell death. Activities of superoxide dismutase SOD and POD, as well as MDA and Pro contents, were measured using commercial assay kits (Greysun Biotech Co., Ltd.) in plants treated with 100 mM and 200 mM NaCl.

### 2.7 Statistical data analysis

All statistical analyses were performed using SPSS 26.0 software, and graphs were generated using Microsoft Excel. Data are expressed as the mean ± standard deviation (SD). Statistical differences were determinedusing one-way analysis of variance (ANOVA) followed by Duncan’s multiple range test (MRT). Differences were considered statistically significant at *P*<0.05. Significant differences between groups are indicated by different lowercase letters (e.g., a, b, c).

## 3 Results

### 3.1 Isolation of *BvKUP13* and sequence analysis

**Figure 1:**
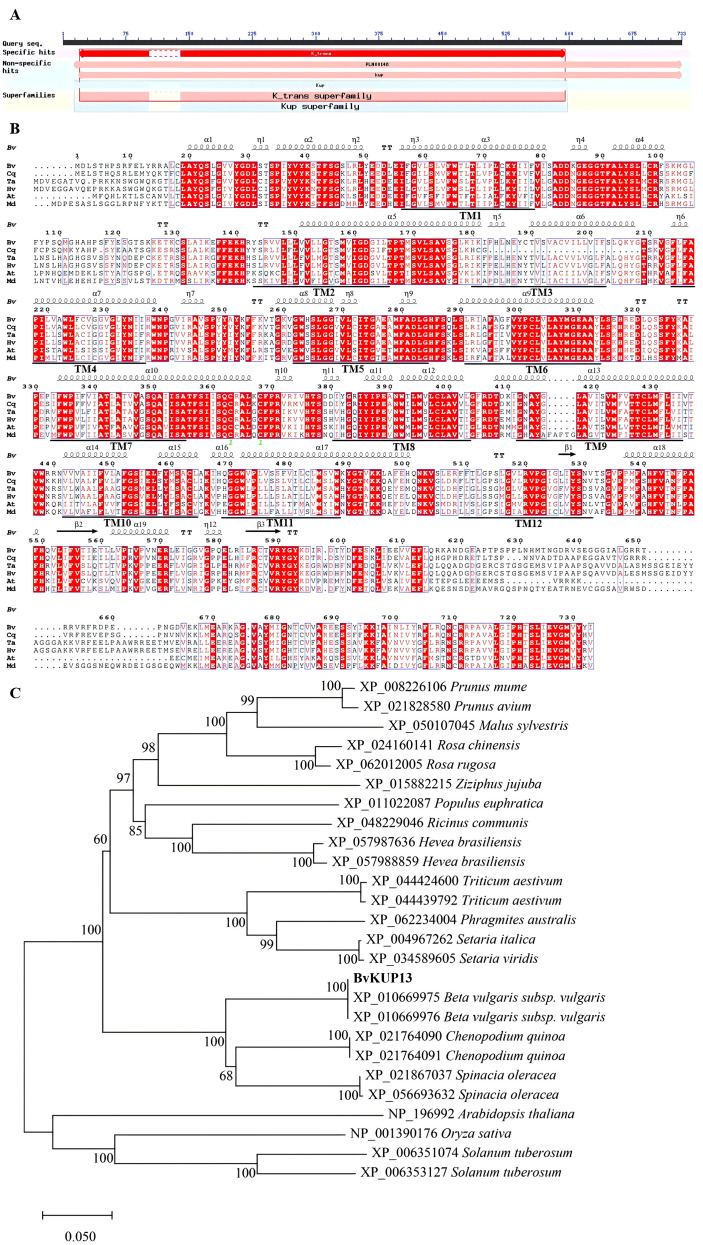
Bioinformatics analysis of BvKUP13. **(A)** Conserved domain architecture of BvKUP13. The protein contains a central K^+^_trans superfamily domain (red bar), indicative of its function as a potassium transporter. **(B)** Multiple sequence alignment of BvKUP13 with homologous plant K^+^ transporters. Identical residues are highlighted in red. Twelve predicted transmembrane helices (TM1–TM12) are marked with black arrows, confirming its typical membrane protein topology. **(C)** Phylogenetic relationship of BvKUP13. The evolutionary tree was constructed with homologs from diverse plant species. BvKUP13 forms a distinct clade with transporters from other Chenopodiaceae species *(Chenopodium quinoa, Spinacia oleracea*), indicating close evolutionary and functional conservation within this family. Bootstrap values (1000 replicates) are shown at nodes. The scale bar represents 0.05 substitutions per site.

In the transcriptomic dataset under salt stress (AK3018), *BvKUP13* expression was significantly increased. The full-length cDNA of *BvKUP13* was obtained using sequencing and RT-PCR. The CDS of *BvKUP13* was 2199 bp long and encoded a 733-amino acid protein, with a predicted molecular weight of 81.76 kDa and an isoelectric point (pI) of 8.96. Sequence alignment with the NCBI database revealed that BvKUP13 contained conserved domains of the K-trans and Kup superfamilies (**Figure 1A**), classifying it as a member of the high-affinity K^+^ transporter family. The predicted secondary structure of the protein consists of 56.81% α-helix, 3.93% β-sheet, 26.31% random coil, and 12.96% extended strand. Transmembrane prediction indicated that the protein possesses 12 transmembrane domains (TM1–TM12). BvKUP13 showed high sequence homology with CqKUP13 from quinoa (90.74%), TaKUP13 from wheat (86.45%), HvKUP13 from barley (86.20%), AtKUP13 from *Arabidopsis* (82.13%), and MdKUP13 from apple (88.40%) (**Figure 1B**). Phylogenetic analysis of amino acid sequences from BvKUP13 in sugar beet and KUP proteins from 16 species, including wheat and *Arabidopsis*, revealed that BvKUP13 clusters with spinach and quinoa proteins, both belonging to the Chenopodiaceae family, indicating close evolutionary relationships (**Figure 1C**).

### 3.2 Subcellular localization analysis of BvKUP13

**Figure 2:**
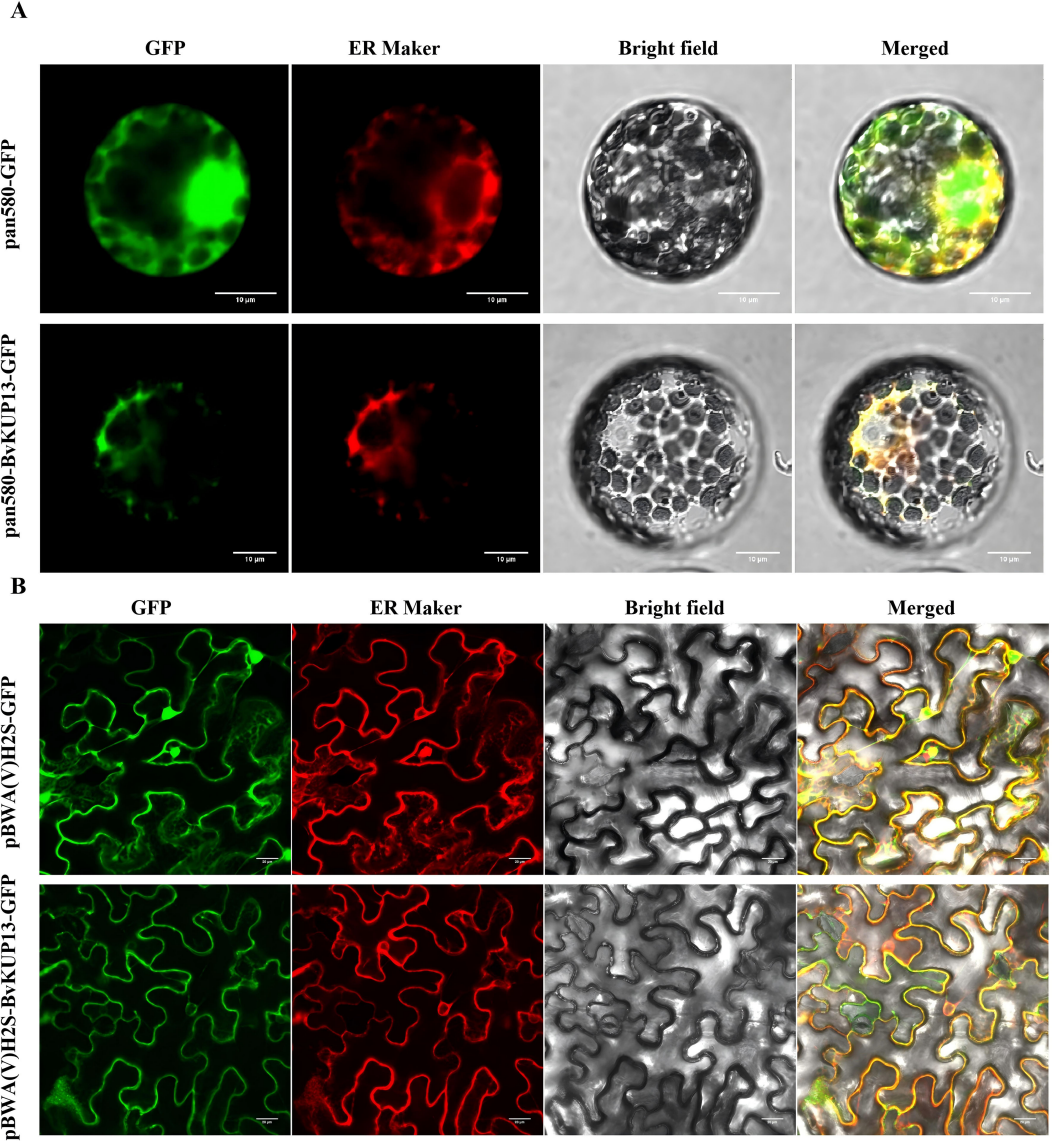
Subcellular localization of BvKUP13. **(A)** Transient expression of pAN580-GFP (empty vector control) and pAN580-BvKUP13-GFP (green) in tobacco protoplasts, co-expressed with an endoplasmic reticulum (ER) marker (red). The green fluorescence of BvKUP13-GFP overlaps with the ER marker signal, indicating ER localization. Scale bar: 10 μm. **(B)** Transient co-expression of pBWA(V)H2S-BvKUP13-GFP (green) and ER marker (red) in tobacco leaf epidermal cells via Agrobacterium-mediated infiltration. Confocal imaging shows a typical reticulate pattern of co-localization. Scale bar: 20 μm.

To investigate the subcellular localization of BvKUP13, transient co-expression assays were performed in two independent *Nicotiana* systems. First, tobacco protoplasts were co-transformed with the pan580-BvKUP13-GFP recombinant vector and an ER red fluorescent marker, using the pan580-GFP empty vector as a negative control. Confocal imaging revealed that the green fluorescence of BvKUP13-GFP co-localized with the red fluorescence of the ER marker, indicating that BvKUP13 is localized to the ER in protoplasts (**Figure 2A**). To further validate this result, the fusion construct pBWA(V)H2S-BvKUP13-GFP and the ER marker were introduced into tobacco leaf epidermal cells via Agrobacterium-mediated infiltration. Confocallaser scanning microscopy showed that the green fluorescence of BvKUP13-GFP displayed a typical reticulate distribution pattern, which overlapped precisely with the red ER marker signal (**Figure 2B**). This co-localization was evident from the merged images, where a strong yellow fluorescence was observed. These findings provide compelling evidence that BvKUP13 is localized to the endoplasmic reticulum.

### 3.3 Analysis of tissue expression characteristics

**Figure 3:**
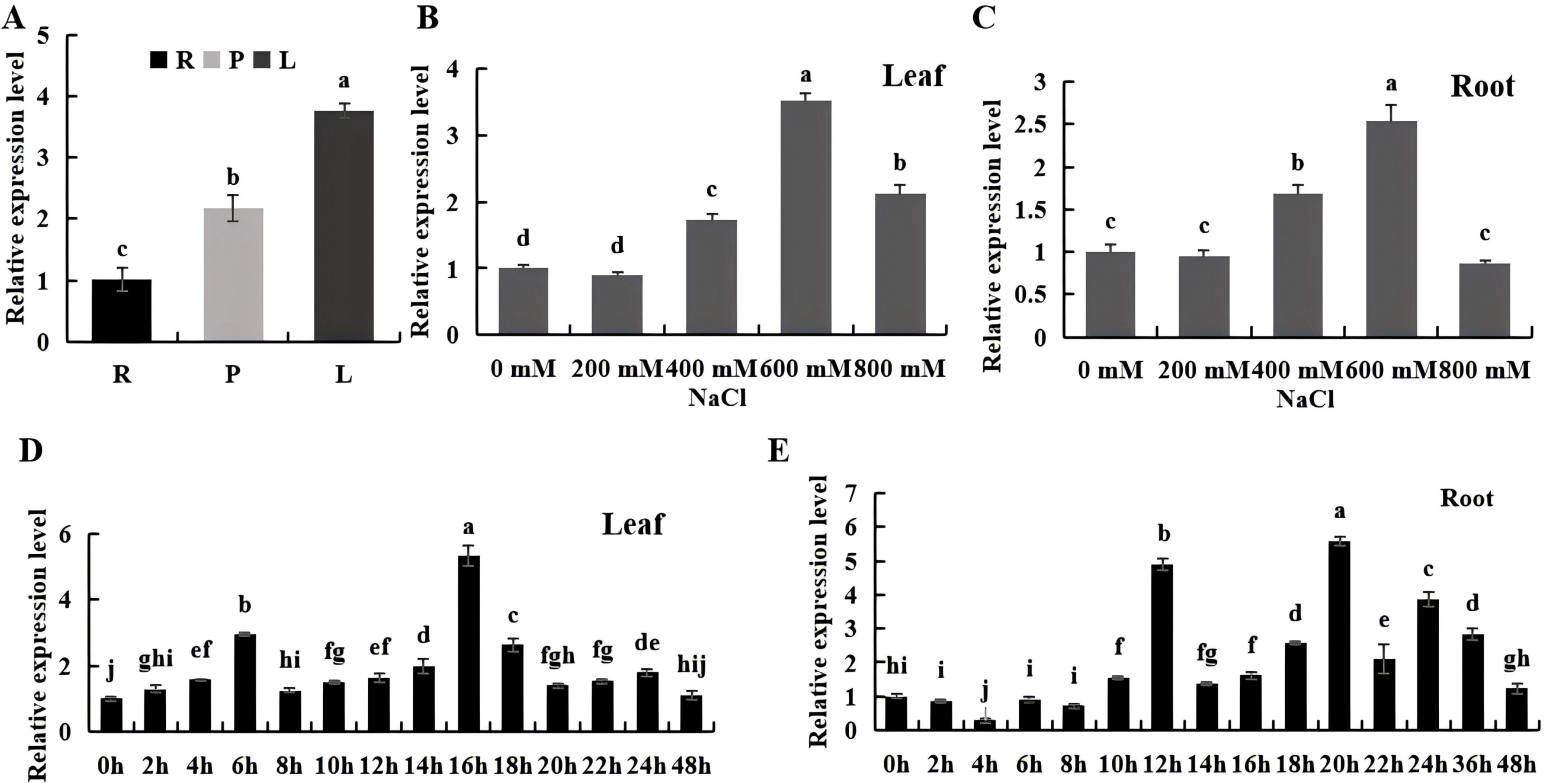
Expression analysis and functional validation of *BvKUPI3* under salt stress. **(A)** Tissue-specific expression of *BvKUPI3* in roots (R), petioles (P), and leaves (L) of sugar beet. Different lowercase letters indicate significant differences (P < 0.05). **(B, C)** NaCl concentration-dependent expression of *BvKUPI3* in leaves **(B)** and roots **(C)** treated with 0–800 mM NaCl for 7 days. Different letters denote significant differences among treatments within each panel (P < 0.05). **(D, E)** Time-course expression of *BvKUPI3* in leaves **(D)** and roots **(E)** under 600 mM NaCl stress over 48 h Different letters indicate significant differences across time points within each panel (P < 0.05). Data information: In panels **(A–E)**, data are presented as mean ± SEM (n = 3 independent biological replicates). Statistical significance (P < 0.05) was determined by one-way ANOVA followed by Duncan’s multiple range test(MRT); groups not sharing a common lowercase letter are significantly different.

To investigate the expression pattern of the *BvKUP13* under salt stress, real-time PCR was used to analyze its expression in the leaves and roots of sugar beet at various NaCl concentrations and time points. Under normal conditions, the *BvKUP13* was mainly expressed in the roots, petiole and leaves, with the highest expression in the leaves and lowest in the roots (**Figure 3A**). Under different NaCl concentrations, the expression of *BvKUP13* in both leaves (**Figure 3B**) and roots (**Figure 3C**) initially increased and then decreased, and expression peaked at 600 mM NaCl. After 48 hours of treatment at this concentration, peak expression in leaves occurred at 16 hours (**Figure 3D**), and in the roots at 20 hours (**Figure 3E**). These results suggested that the *BvKUP13* can be significantly induced by salt stress.

### 3.4 Identiffcation and screening of *BvKUP13*-overexpressing transgenic *Arabidopsis*

**Figure 4:**
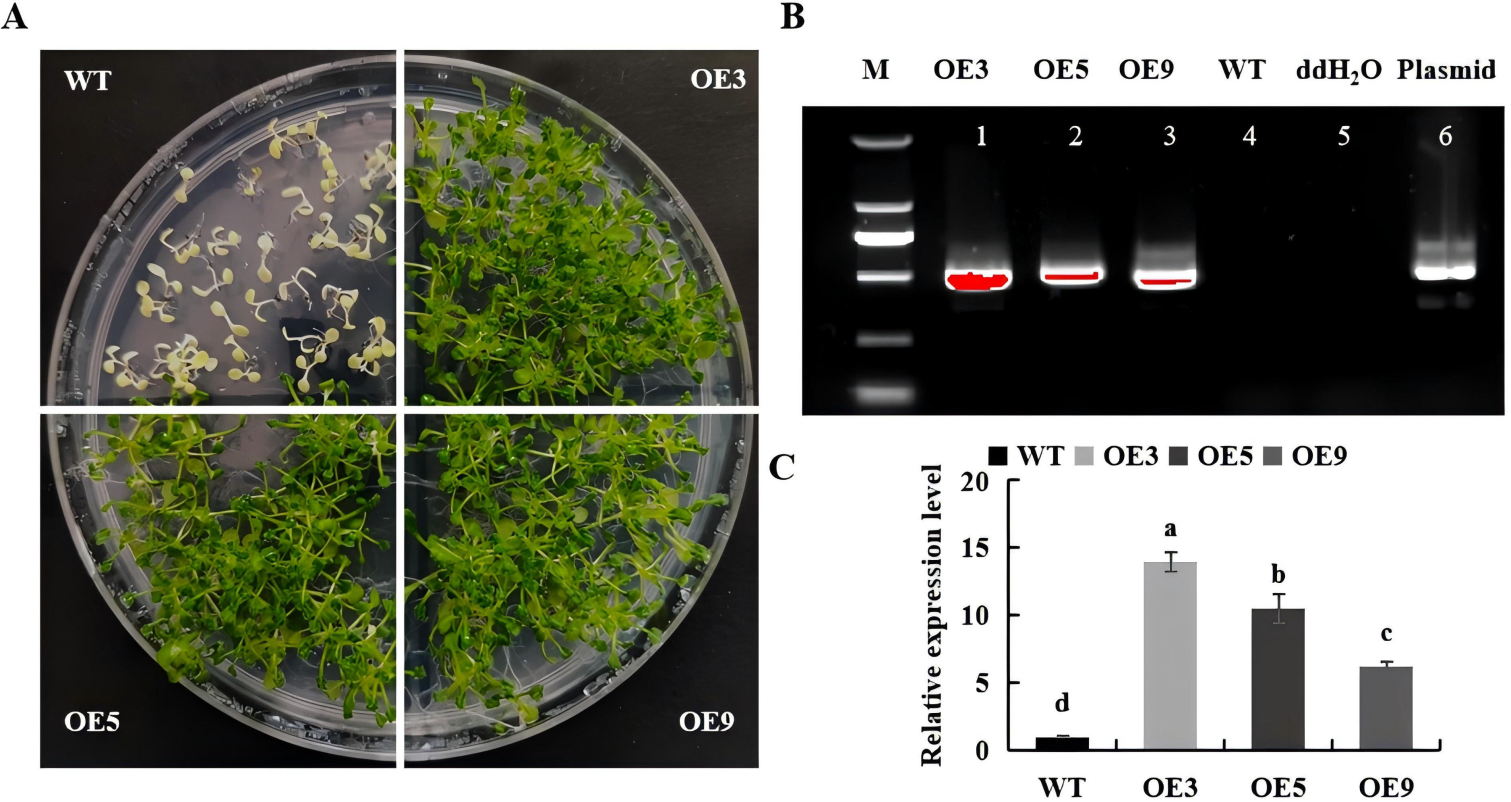
Molecular identification of *BvKUP13*-transformed *A thaliana* lines. **(A)** Selection of homozygous transgenic lines. T_3_ seedlings were grown on 1/2 MS medium containing 50 mg L^−1^ kanamycin for 10 days. **(B)** PCR-based genotyping. Genomic DNA was amplified with *BvKUPI3*-specific primers. Lanes: M, DNA ladder; 1–3, independent overexpression lines (OE3, OE5, OE9); 4, wild-type (WT) control; 5, no-template control (ddH_2_O); 6, plasmid DNA positive control. **(C)** Relative expression levels of *BvKUP13* in transgenic lines. Different lowercase letters indicate statistically significant differences among lines (P < 0.05). Data information: In **(C)**, values represent mean ± SEM of three biological replicates. Statistical analysis was performed using one-way ANOVA followed by Duncan’s multiple range test (P < 0.05).

The pBI101-*BvKUP13* overexpression vector was constructed, and WT was genetically transformed using the Agrobacterium-mediated floral dip method. Homozygous transgenic lines were obtained through self-pollination. Three resistant lines (OE3, OE5,and OE9) were randomly selected and screened for kanamycin resistance (50 mg/L) through the T_3_ generation (**Figure 4A**). Genomic PCR analysis confirmed the insertion of the foreign gene into the genomes of these plants (**Figure 4B**). Real-time PCR analysis revealed that the expression of *BvKUP13* in transgenic *Arabidopsis* was significantly higher than in WT plants, with varying expression levels among different lines (**Figure 4C**), confirming the successful generation of transgenic *Arabidopsis*.

### 3.5 Overexpression of *BvKUP13* enhanced the salt tolerance in *Arabidopsis*

**Figure 5:**
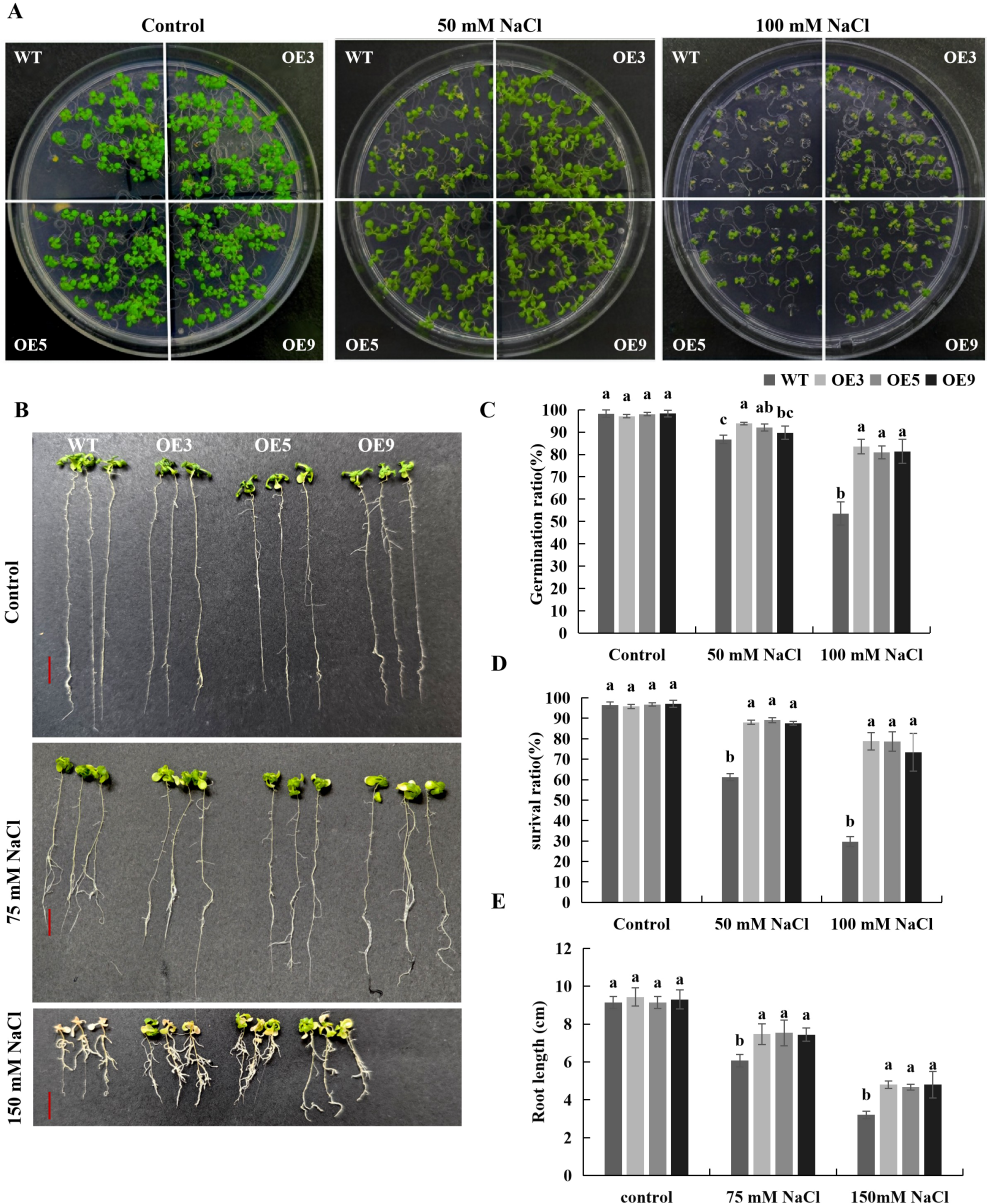
Seedlings Phenotype of WT and *BvKUP13*-overexpressing transgenic *Arabidopsis* plants under NaCl stress. **(A)** Shoot growth phenotype under salt stress. Seedlings of wild-type (WT) and three independent overexpression lines (OE3, OE5, OE9) were grown on MS plates for 14 days under control, 50 mM, or 100 mM NaCl conditions. Overexpression lines maintained greener and larger rosettes under stress. **(B)** Root growth phenotype under salt stress. Representative images show the root systems of WT and overexpression lines grown on control, 75 mM, or 150 mM NaCl for 7 days. Overexpression lines developed longer and more extensive roots under NaCl treatment. **(C)** Germination rate. Percentage of germinated seeds was scored after 7 days on medium containing 0, 50, or 100 mM NaCl. Different lowercase letters indicate significant differences among genotypes within each treatment (P < 0.05). **(D)** Survival rate. Survival was assessed after 14 days of salt treatment (0, 50, or 100 mM NaCl). Different letters denote significant differences among genotypes within each treatment (P < 0.05). **(E)** Primary root length. Root length was measured after 7 days of growth on medium with 0, 75, or 150 mM NaCl. Different letters indicate significant differences among genotypes within each treatment (P < 0.05). Data information: In **(C–E)**, data are presented as mean ± SEM (n = 3 independent biological replicates, each with ≥20 plants/seedlings). Statistical significance was determined by one-way ANOVA followed by Duncan’s multiple range test(MRT) (P < 0.05). Groups not sharing a common lowercase letter within the same NaCl concentration are significantly different.

To examine the impact of salt stress on the growth of transgenic *Arabidopsis*, seeds were planted in MS medium containing varying NaCl concentrations, and the plant phenotypes were observed. The results indicated no significant difference between WT and transgenic *Arabidopsis* under normal conditions. Under salt stress, however, the seed germination rate and seedling survival rate of transgenic *Arabidopsis* overexpressing *BvKUP13* were significantly higher than those of WT (**Figures 5A, C, D**). After 7 days of vertical growth, no significant difference in root length was observed between the WT and transgenic *Arabidopsis*. However, under different NaCl concentration conditions, the root length of the transgenic *Arabidopsis* was significantly greater than that of the WT (**Figures 5B, E**). This suggested that *BvKUP13* may be involved in significantly improving the germination rate, survival rate and root development of seedlings under salt stress.

**Figure 6:**
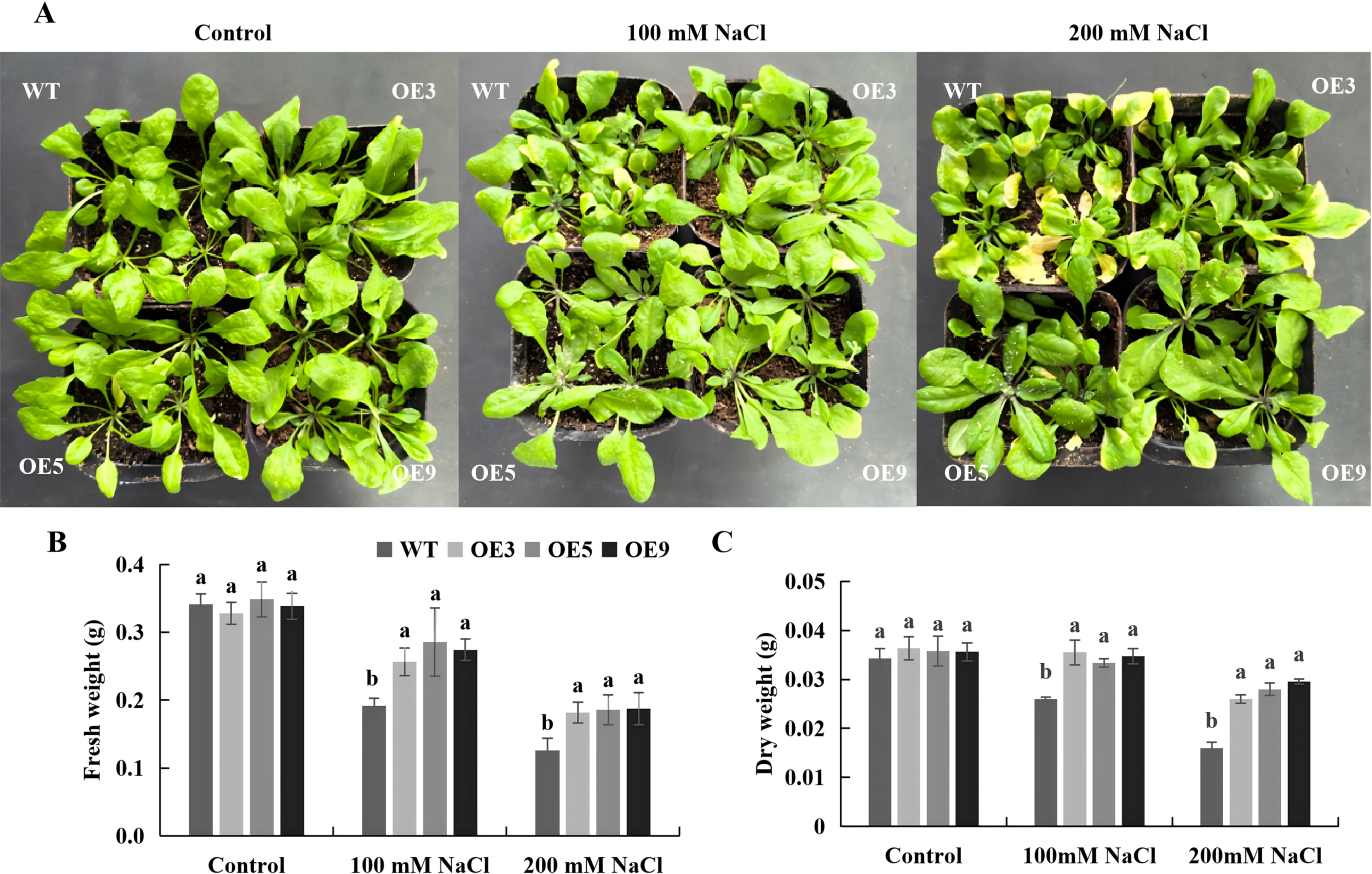
Leaf and plant growth of WT and *BvKUP13*-overexpressing transgenic *Arabidopsis* plants under NaCl stress. **(A)** Representative plant phenotypes. Four-week-old soil-grown plants of wild-type (WT) and three overexpression lines (OE3, OE5, OE9) were irrigated with 0, 100, or 200 mM NaCl solution. Overexpression lines displayed less leaf chlorosis and wilting under stress. **(B)** Fresh weight per plant. Above ground tissues were harvested and weighed immediately after the 7-day treatment. Different lowercase letters indicate significant differences among genotypes within each treatment (P < 0.05). **(C)** Dry weight per plant. The same tissues were oven-dried to constant weight before measurement. Different letters denote significant differences among genotypes within each treatment (P < 0.05). Data information: In **(B, C)** data are presented as mean ± SEM (n = 3 independent biological replicates). Statistical significance was determined by one-way ANOVA (P < 0.05). Groups not sharing a common letter within the same NaCl concentration are significantly different.

Transgenic *Arabidopsis* and WT plants grown in soil for 30 d were subjected to various NaCl concentrations. No significant difference was observed in the growth between WT and transgenic plants under normal conditions. However, under salt stresses, both WT and overexpressing lines exhibited growth inhibition and leaf chlorosis, though the inhibition was more pronounced in the WT (**Figure 6A**). The dry and fresh weights of WT plants were significantly lower than those of the overexpressing lines (**Figures 6B, C**). These results suggested that overexpression of *BvKUP13* significantly enhanced the salt tolerance of transgenic *Arabidopsis*.

### 3.6 Transgenic *Arabidopsis* overexpressing *BvKUP13* exhibited better photosynthesis under salt stress

**Figure 7:**
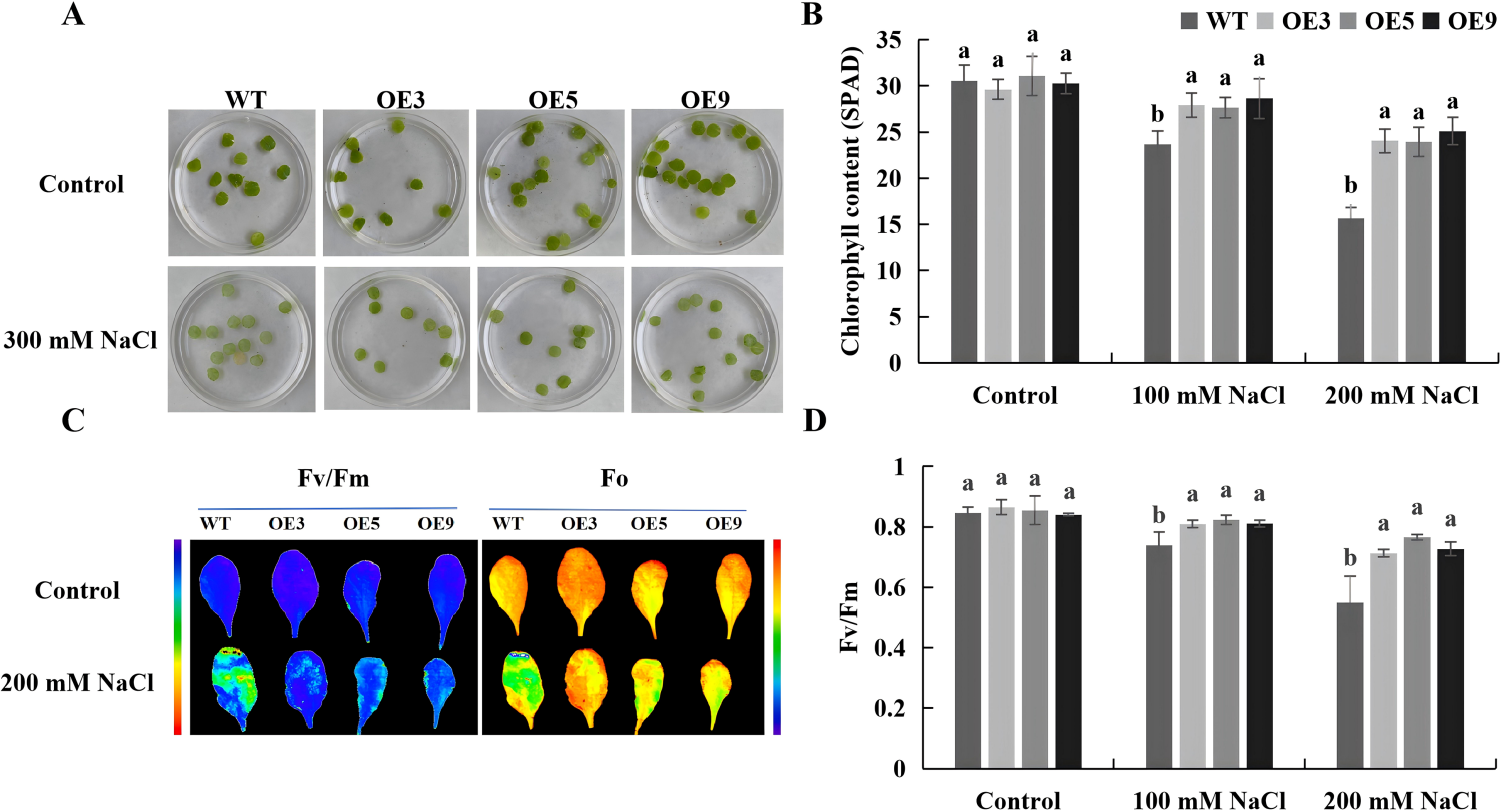
photosynthesis of WT and *BvKUP13*-overexpressing transgenic *Arabidopsis* plants under NaCl stress. **(A)** Leaf disc senescence assay. Detached leaf discs (1-cm diameter) from 4-week-old plants were incubated in 300 mM NaCl solution in the dark for 5 days to accelerate stress-induced senescence. **(B)** Leaf chlorophyll content. SPAD values were measured from rosette leaves of soil-grown plants irrigated once with 0, 100, or 200 mM NaCl solution and measured 7 days later. Different lowercase letters indicate significant differences among genotypes within each treatment (P < 0.05). **(C)** Chlorophy ll fluorescence imaging. False-color images represent the maximum quantum efficiency of PSII (Fv/Fm) in leaves of plants under control or after a single irrigation with 200 mM NaCl, imaged 7 days post-treatment. Warmer colors indicate higher photochemical efficiency. **(D)** Quantitative analysis of PSII efficiency (Fv/Fm). Fv/Fm values were quantified from plants irrigated once with 0, 100, or 200 mM NaCl and measured after 7 days. Different letters denote significant differences among genotypes within each treatment (P < 0.05). Data information: In **(B)** and **(D)** data are presented as mean ± SEM (n =3 independent biological replicates). Statistical significance was determined by one-way ANOVA followed by Duncan’s multiple range test(MRT) (P < 0.05). Groups not sharing a common lowercase letter within the same NaCl concentration are significantly different.

To investigate the role of *BvKUP13* in regulating the photosynthetic system under salt stress, we analyzed leaf disc senescence, chlorophyll content, and photosystem II (PSII) activity in both transgenic and WT plants. The results indicated that in the control group, both transgenic *Arabidopsis* and WT plants exhibited similar growth, with no significant differences. Under salt stress, the overexpressing transgenic *Arabidopsis* displayed lower leaf disc senescence and significantly higher chlorophyll content compared to WT (**Figures 7A, B**). The overexpressing lines exhibited stronger chlorophyll fluorescence under 200 mM NaCl stress compared to WT (**Figure 7C**), which was consistent with the Fv/Fm measurement results (**Figure 7D**). Chlorophyll content is positively correlated with photosynthetic efficiency within a certain range, suggesting that *BvKUP13* enhances photosynthetic activity by increasing chlorophyll content under salt stress.

### 3.7 Overexpression of *BvKUP13* enhanced the accumulation of K^+^ and maintained the lower Na^+^/K^+^ ratio in transgenic plants

**Figure 8:**
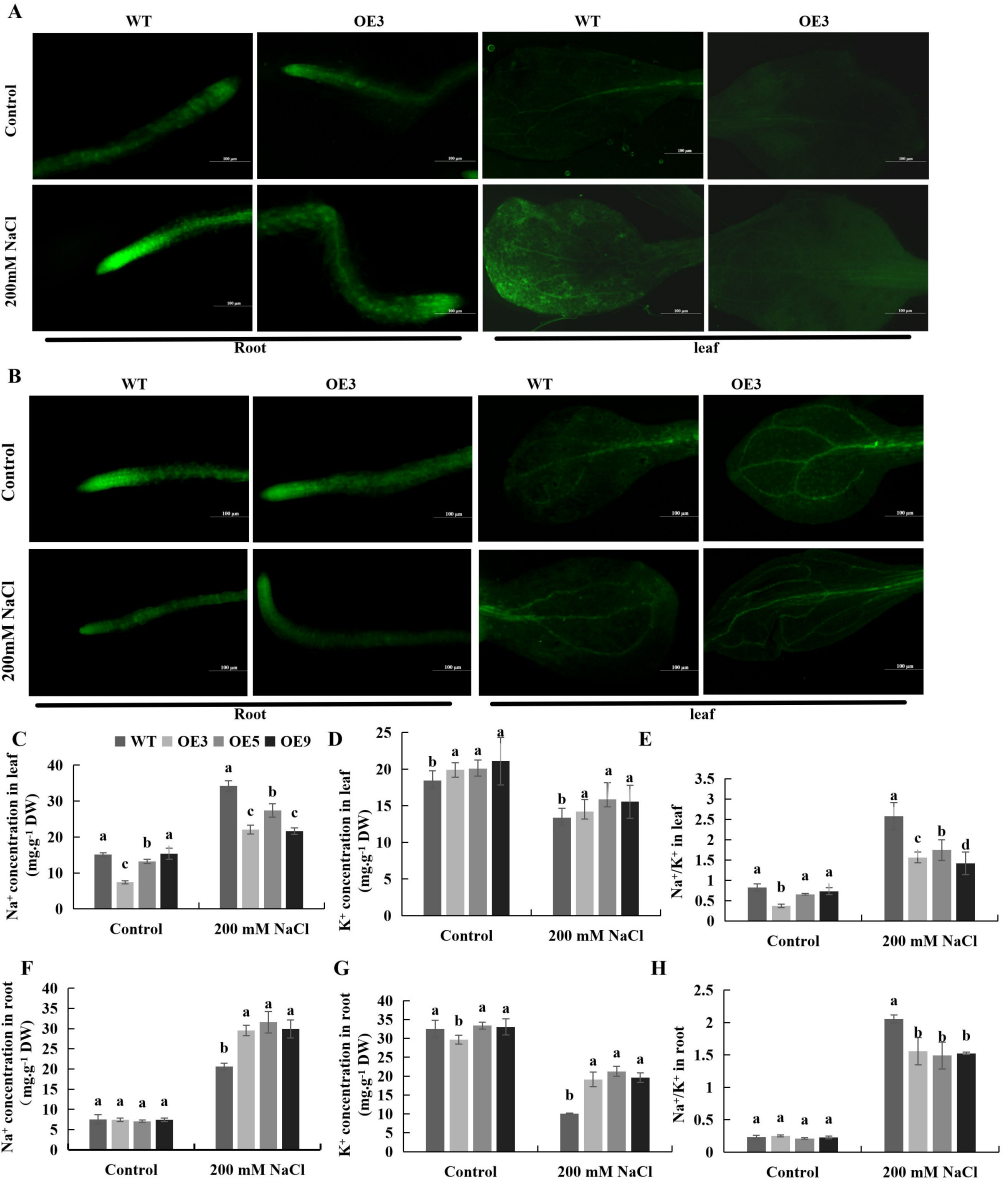
Distribution of Na^+^ and K^+^ of WT and *BvKUP13*-overexpressing transgenic *Arabidopsis* plants under NaCl stress. **(A)** Na^+^ fluorescent staining in roots and leaves. Ten-day-old seedlings were transferred to medium containing 200 mM NaCl for 3 days prior to staining (scale bar = 100 μm). Green fluorescence indicates Na^+^ accumulation. Overexpression lines show reduced fluorescence intensity compared to WT. **(B)** K^+^ fluorescent staining in roots and leaves. Seedlings were treated as in **(A)** and stained for K^+^ (scale bar = 100 μm). Overexpression lines maintain stronger fluorescence, indicating higher K^+^ retention. **(C–E**) Ion content in roots. Na^+^ content **(C)**, K^+^ content **(D)**, and the Na^+^/K^+^ ratio **(E)** were measured in roots of soil-grown plants irrigated with 200 mM NaCl for 7 days. Different lowercase letters indicate significant differences among genotypes (P < 0.05). **(F–H)** Ion content in leaves. Na^+^ content **(F)**, K^+^ content **(G)**, and the Na^+^/K^+^ ratio **(H)** were measured in leaves of the same plants as in **(C–E)**. Different letters denote significant differences among genotypes (P < 0.05). Data information: In **(C–H)**, data are presented as mean ± SEM (n = 3 independent biological replicates). Statistical significance was determined by one-way ANOVA followed by Duncan’s multiple range test (P < 0.05). Groups not sharing a common lowercase letter are significantly different.

To examine the role of *BvKUP13* in regulating ion balance under salt stress in transgenic *Arabidopsis*, the contents of Na^+^ and K^+^, along with their distribution in the roots and leaves of transgenic lines and WT, were analyzed. Under normal conditions, Na^+^ fluorescence signals in both the roots and leaves of all lines were weak, with no significant differences. Under salt stress conditions, the Na^+^ fluorescence signal was significantly enhanced in the root apex and elongation zone, as well as in mesophyll cells and around the leaf veins of both WT and *BvKUP13*-overexpressing *Arabidopsis* (**Figure 8A**), the contents of Na^+^ were significantly higher in both the leaves (**Figure 8C**) and roots (**Figure 8F**), but Na^+^ fluorescence intensity and content in transgenic plants were significantly lower than in WT. Under normal conditions, both WT and overexpressing lines exhibited strong, evenly distributed K^+^ fluorescence signals in their roots and leaves. However, K^+^ fluorescence intensity in WT significantly decreased compared to overexpressing lines under salt stress conditions (**Figure 8B**). Additionally, K^+^ content in both the leaves (**Figure 8D**) and roots (**Figure 8G**) was significantly reduced, with the roots exhibiting a more sensitive response. Under salt stress, the Na^+^ content in the leaves of transgenic plants was significantly lower than in WT, while the K^+^ content remained unchanged. This led to a substantial reduction in the Na^+^/K^+^ ratio in transgenic plants compared to WT (**Figure 8E**). In contrast, both Na^+^ and K^+^ contents in the roots of transgenic plants were significantly higher than in WT, resulting in a markedly lower Na^+^/K^+^ ratio (**Figure 8H**). These findings suggested that *BvKUP13* enhanced potassium ion uptake, reduced sodium ion accumulation, and maintained a low Na^+^/K^+^ ratio, thereby improving the salt tolerance of *Arabidopsis*. Furthermore, *BvKUP13* may play a more prominent role in ion transport within the roots, limiting the transport of sodium ions to the shoot.

### 3.8 Overexpression of *BvKUP13* improved antioxidant and osmotic adjustment abilities of transgenic plants under salt stress

**Figure 9:**
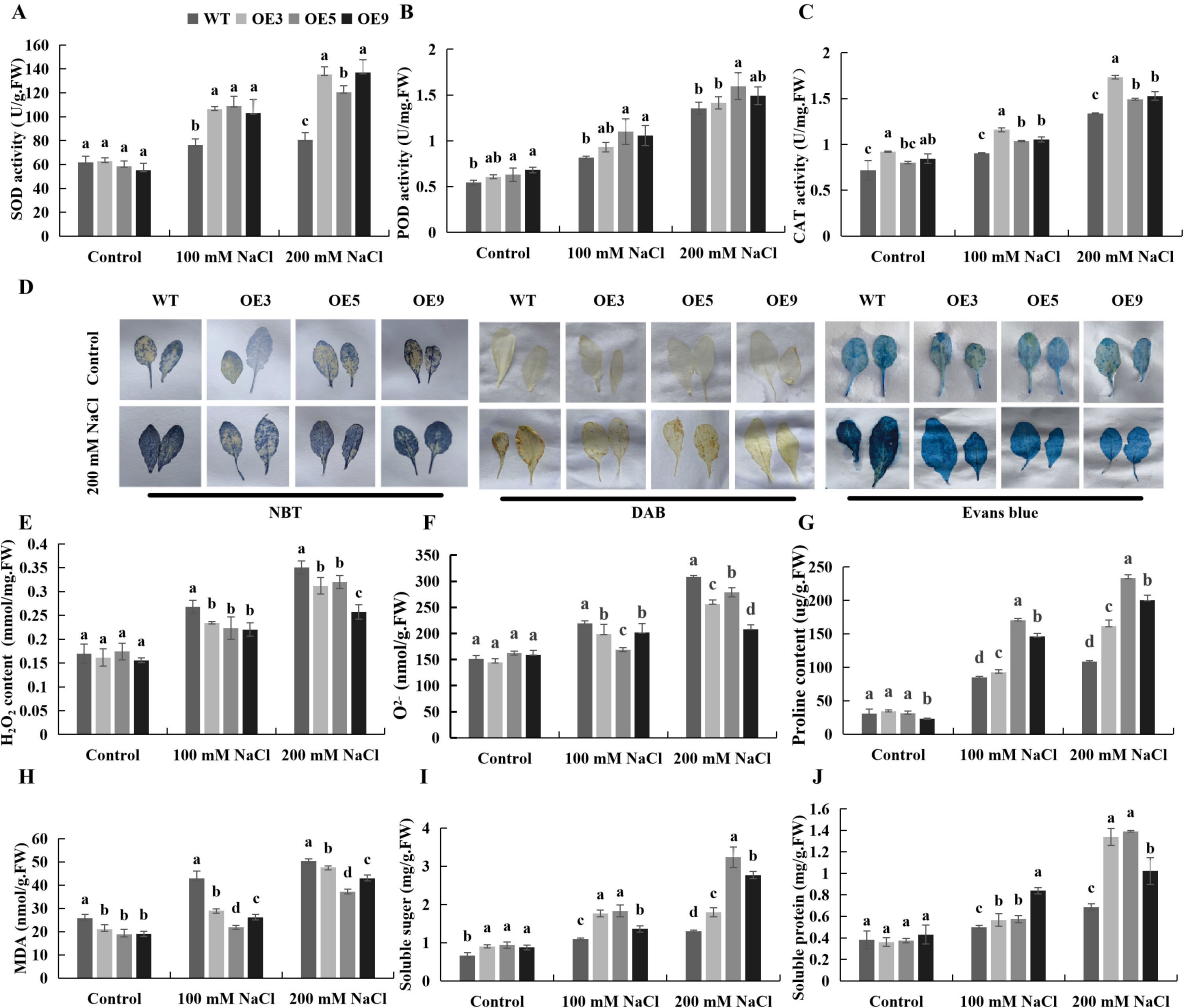
Determination of resistance physiological of WT and *BvKUP13*-overexpressing transgenic *Arabidopsis* plants under NaCl stress. **(A–C)** Activities of key antioxidant enzymes. Superoxide dismutase (SOD, **(A)**), peroxidase (POD, **(B)**), and catalase (CAT, **(C)**) activities were measured in rosette leaves of soil-grown plants irrigated once with 0, 100, or 200 mM NaCl and harvested 7 days later. Different lowercase letters indicate significant differences among genotypes within each treatment (P < 0.05). **(D)** Histochemical detection of oxidative stress and cell death. Leaves from plants treated with 200 mM NaCl for 7 days were stained with nitro blue tetrazolium (NBT, for O_2_^−^), 3,3′-diaminobenzidine (DAB, 90iokjfor H_2_O_2_), and Evans blue (for dead cells). Overexpression lines show visibly weaker staining. **(E–J)** Quantification of stress physiological markers. Hydrogen peroxide (H_2_O_2_, **(E)**), superoxide anion (O_2_^−^, **(F)**), proline **(G)**, malondialdehyde (MDA, **H**), soluble sugar **(I)**, and soluble protein **(J)** contents were determined in leaves from the same plants as in **(A–C)**. Different letters denote significant differences among genotypes within each treatment (P < 0.05). Data information: In **(A–C, E–J)**, data are presented as mean ± SEM (n = 3 independent biological replicates). Statistical significance was determined by one-way ANOVA followed by Duncan’s multiple range test (P < 0.05). Groups not sharing a common lowercase letter within the same NaCl concentration are significantly different.

To further investigate how the *BvKUP13* enhanced plant tolerance to salt stress, the activity of the ROS scavenging system and the accumulation of osmotic regulators were examined under stress conditions. Under normal conditions, no significant differences were observed in the levels of antioxidants and osmotic regulators between transgenic plants and WT. As NaCl concentration increased, the activities of SOD, POD, and CAT (**Figures 9A–C**) in transgenic plants were significantly higher than in WT, while H_2_O_2_ and O_2_^-^ (**Figures 9E, F**) levels were significantly lower in the transgenic plants. These findings were consistent with the NBT and DAB (**Figure 9D**) staining results. After salt stress, Evans Blue staining was more intense in WT, indicating greater cell damage (**Figure 9D**). MDA accumulation was significantly higher in the WT, indicating greater lipid peroxidation of the membrane (**Figure 9H**). Proline (**Figure 9G**), soluble sugars (**Figure 9I**), and soluble proteins (**Figure 9J**), which serve as osmotic regulators, were significantly more abundant in the overexpressing lines under high-salt conditions than in the WT. These results suggested that *BvKUP13* overexpression enhanced the efficiency of the ROS scavenging system in *Arabidopsis* under salt stress conditions, promoted osmotic regulator accumulation, and improved salt tolerance.

## 4 Discussion

Members of the HAK/KUP/KT transporter gene family have been cloned from various plants, contained conserved domains of the potassium transporter superfamily, and were presented on the membranes of various organelles, and exhibits a broad tissue expression pattern in plants, suggesting that they may perform diverse cellular and physiological functions. The prediction for the BvKUP13 protein suggested the presence of 12 transmembrane domains and conserved domains from the K-trans and Kup superfamilies, exhibiting similar physicochemical properties to homologous proteins found in plants such as banana (Liu S. et al., 2022) and grape (Lei et al., 2022). Similarly, *TaHAK13* (Run et al., 2022), *TaHAK18* (Liu T. et al., 2025), and *AtKUP7* (Han et al., 2016) were located on the plasma membrane, whereas *AoKUP2* and *AtKUP2* are localized to the plasma membrane and mitochondria (Rajappa et al., 2020). Further research confirmed that *AtKUP9* is primarily localized in the endoplasmic reticulum (ER), and it mediates the efflux of potassium and auxin from the ER lumen to the cytoplasm of quiescent center cells at the root tip under low K^+^ conditions (Zhang et al., 2020). In this work, the localization of *BvKUP13* in the ER suggested that it may regulate the K^+^ concentration within the ER, thereby balancing the ionic gradient between the ER and the cytoplasm. Previously study showed that KUPs were induced by high Na^+^ or low K^+^ conditions, such as that in cotton (*Gossypium hirsutum*) (Wang et al., 2022), black goji berry (Wei et al., 2022), and pepper (Zhao et al., 2022), For instance, among the 15 *FveKUP* genes in strawberry, 8 exhibited significantly higher expression in flowers, *FveKUP4* is highly expressed in roots, *FveKUP12* in leaves, while *FveKUP3*, *FveKUP6*, *FveKUP9*, *FveKUP11*, and *FveKUP13* were not detected in any of the measured organs (Gao et al., 2021). *BvKUP13* was highly expressed in both the above-ground and below-ground parts of the plant, with expression significantly up regulated under salt stress. This suggests its role in ion absorption in the roots and long-distance transport to the leaves, implying that *BvKUP13* helps maintain whole-plant ion homeostasis through tissue-specific regulation.

Previous studies have demonstrated that the *KUP/HAK/KT* may improve plant salt tolerance by enhancing root biomass and survival rates. The *PeHAKs* in *Phyllostachys edulis* played roles in tissue and organ development, promoted rapid growth, and facilitate K^+^ ion transport under various abiotic stress conditions (Liu J. et al., 2022). *TaHAK18* significantly increased plant fresh weight and root length and promotes lateral root development under both low and normal K^+^ conditions (Liu T. et al., 2025). The *Arabidopsis atkup12* mutant showed reduced germination rates under salt stress, impaired cotyledon greening under oxidative stress, and shorter root lengths (Zhang et al., 2021). *Arabidopsis* overexpressing the *HcKUP12* from *Halostachys caspica* showed improvements in root length and shoot weight, compared to WT under NaCl stress (Zhang S. et al., 2022). This study found that transgenic lines overexpressing *BvKUP13* exhibited higher germination and survival rates, increased root lengths, fresh weights, dry weights, and overall better growth under salt stress compared to WT. *BvKUP13* alleviated salt stress-induced damage to the photosynthetic apparatus by increasing chlorophyll content and protecting chloroplast structure integrity under salt stress. Thus, overexpression of *BvKUP13* reduces growth inhibition and suppresses photosynthesis under salt stress, thereby enhanced the plant salt tolerance.

Maintaining a stable intracellular K^+^ concentration during salt stress is essential for plants to alleviate salt-induced damage. Plants achieve this by accumulating K^+^, which helps maintain a stable K^+^/Na^+^ balance. For example, under both K^+^-sufficient and K^+^-deficient conditions, *Arabidopsis atnhx1–1* mutants expressing *ZxNHX1* exhibited enhanced Na^+^ sequestration into vacuoles and improved K^+^ retention in the cytosol. At the whole-plant level, *ZxNHX1* controlled Na^+^ uptake in roots and facilitated Na^+^ transport to leaves. This reprogrammed ion distribution promoted energy-efficient osmotic adjustment, ultimately strengthening stress tolerance and enhancing biomass compared to *AtNHX1* expressing-plants (Liu H. S. et al., 2025). As high-affinity K^+^ transporters, members of the KUP/HAK/KT family perform diverse functions under stress conditions across various species. For instance, overexpression of *OsHAK5* in rice enhanced K^+^ uptake under low K^+^ conditions and played a key role in root-to-shoot K^+^ translocation (Yang et al., 2014). In contrast, overexpression of *OsHAK2* increased salt sensitivity but promoted shoot growth under low Na^+^ and K^+^ conditions in rice (Morita et al., 2023). Compared to WT, the *oshak18* mutant accumulated more K^+^ in stems and less in roots under low K^+^ conditions, resulting in an increased stem-to-root K^+^ ratio. These findings suggested that *OsHAK18* mediates K^+^ loading and redistribution in the phloem, and its disruption promoted K^+^ retention in stems under low K^+^ conditions (Alhasnawi1 et al., 2014). This study found that *BvKUP13* not only regulated ion uptake in roots but also contributed to ion homeostasis in aerial tissues. Under salt stress, overexpression lines exhibited lower Na^+^ levels and higher K^+^ levels in both roots and leaves compared to WT, leading to a lower Na^+^/K^+^ ratio. Histochemical staining further showed that *BvKUP13*-overexpressing plants exhibited enhanced Na^+^ efflux and increased K^+^ accumulation in roots. Additionally, significant K^+^ enrichment was observed in leaf veins, suggesting that *BvKUP13* promoted K^+^ redistribution to maintain cytosolic K^+^ homeostasis in mesophyll cells, thereby supporting stomatal movement and enhancing photosynthetic efficiency. Taken together with previous findings, we propose that *BvKUP13* enhances salt tolerance in transgenic *Arabidopsis* through coordinated root–shoot regulation, which collectively reduces the Na^+^/K^+^ ratio. The marked improvement in root growth observed in transgenic *Arabidopsis* suggests that *BvKUP13* plays a critical role in root-mediated salt stress resistance. Although *BvKUP13* expression is relatively low in beet roots under normal conditions, its salt-induced up regulation may be sufficient to confer protective effects. The ability of roots to sustain growth under high sodium stress, even when aerial tissues are damaged, indicates that enhanced salt tolerance may result from restricted sodium transport, likely mediated by root-localized *BvKUP13* activity. These findings provide a solid foundation for future in-depth functional analyses of *BvKUP13*.

Under stressful conditions, as reactive oxygen species (ROS) accumulate within the plant, antioxidant enzymes increase their activity to eliminate harmful substances and protect the cell membrane. Additionally, osmoregulatory substances also influence the production and clearance of ROS (Alhasnawi1 et al., 2014). The overexpression of *CeqHAK6* and *CeqHAK11* in *Casuarina equisetifolia* (Wang et al., 2023) could enhance the activity of antioxidant enzymes (CAT, POD, and SOD), thereby reducing ROS and MDA accumulation and improving salt tolerance. Similar to the results that have been reported, under salt stress, transgenic *Arabidopsis* overexpressing *BvKUP13* exhibited higher activities of protective enzymes such as SOD, POD, and CAT than those in WT, while superoxide anion and hydrogen peroxide levels were significantly lower than those in WT. This suggests that *BvKUP13* may indirectly activate the antioxidant enzyme defense network by reducing Na^+^-induced membrane lipid peroxidation and ROS accumulation. The overexpression of the *IbHAK11* (Zhu et al., 2023), *MeHKT1* (Luo et al., 2024), and *AtKUP9* (Šustr et al., 2024) in *Arabidopsis* led to higher levels of soluble sugars, proline, and other osmoregulatory substances compared to the WT. The increase in osmotic substances helped maintain the osmotic balance between the inside and outside of the cell, thereby enhancing salt tolerance. In this study, as salt concentration increased, the levels of proline, soluble sugars, and soluble proteins all showed an upward trend, but their contents in the transgenic plants were significantly higher than those in WT. However, as the salt concentration increased, the soluble sugar and soluble protein contents in transgenic *Arabidopsis* showed a substantial increase, while in WT, salt stress may have surpassed its tolerance threshold, leading to severe cell damage, so under high salt conditions, the increase in these contents tended to plateau. This suggests that the transgenic *Arabidopsis* has enhanced osmoregulatory ability, which improves its salt tolerance. Meanwhile, the synthesis of osmoregulatory substances may be regulated by ion homeostasis mediated by *BvKUP13*.

## 5 Conclusions

This study conducted a preliminary analysis of the expression patterns and salt tolerance function of the *BvKUP13*. The results indicated that *BvKUP13* expression was strongly induced by salt stress, and its encoded protein was localized in the endoplasmic reticulum. In *Arabidopsis*, *BvKUP13* decreased the Na^+^/K^+^ ratio by limiting Na^+^ influx and promoting K^+^ uptake. It also activated the antioxidant defense and osmotic regulation systems, thereby enhancing the resistance of transgenic plants to high salinity. This work provided a foundation for future functional studies of the *BvKUP13*.

In conclusion, the native promoter of *BvKUP13* should be employed to elucidate its precise spatial and temporal expression pattern in sugar beet. Moreover, *BvKUP13* represents a promising genetic target for breeding programsaimed at enhancing salt tolerance in sugar beet.

## Data Availability

The gene sequences are publicly available in the NCBI repository, accession number [XM010671674.3]. Further inquiries can be directed to the corresponding author.
