# Supplementary material for: Overexpression of BvKUP13 from sugar beet increased salt tolerance in transgenic Arabidopsis thaliana
Source: Front Plant Sci. 2026 Feb 23;17:1736699. doi: 10.3389/fpls.2026.1736699 (PMC12970625; doi:10.3389/fpls.2026.1736699)
Supplement: Supplementary file 1 [file DataSheet1.zip › Supplement-Wang-Experimental Data/Phylogenetic tree sequence.docx]

#mega

!Title a;

!Format DataType=protein;

#BvKUP13

GTFALYSLLCRFSKMGLFYPSQMGHAHPSFYESGTSKKETKCSL---AIKEFFEKHRYSRVVLLLVVLLGTSMVIGDGILTPTMSVLSAVSGLKIKIP-HLNEYCTVSVACVILLVIFSLQKYGTSRVGFLFAPILVAWLFCVGGVGLYNIIHWNPGVIRAVSPYYAYKFFKVTGKVGWHSLGGIVLCITGAEAMFADLGHFSQLSLRIAFAGFVYPCLVLAYMGEAAYLSEH---REDLQSSFYKAIPEPIFWPIFVIATLATVVASQAIISATFSIISQCRALKCFPRVRIVHTSDDIYGRIYIPEANWTLMVLCLAVVLGFRDTDKIGNAYGLAVISVMFVTTCLMFLIIVTVWRRNVVVAIIFVLAFGSIELFYMSACLAKIHQGGWVPLVSSFVILCLMSVWKYGTVKKLAFEHQNKVSLERFFTL--GPSLGVLRVPGIGLIYSNVTSGVPPMFAHFVTNFPAFHQVLIFVTIETLLVPTVPVNERLEIGGVGPQELRILRCTVRYGYKDIR-DTYDFESKLIEEVVEFLQRK-----AND-------GEAPTPSPPLNHM---T---------------NGDRVSEGGGIALGRRTRRV-RFRD-P------------EPNGDVEKLMEARKAG-VAYMIGNTCVVAREESSYIKKIAINLIYRFLRQNCRRPAVALGIPHTSLIEVGMVYYI

#XP_010669976_Beta_vulgaris_subsp._vulgaris

GTFALYSLLCRFSKMGLFYPSQMGHAHPSFYESGTSKKETKCSL---AIKEFFEKHRYSRVVLLLVVLLGTSMVIGDGILTPTMSVLSAVSGLKIKIP-HLNEYCTVSVACVILLVIFSLQKYGTSRVGFLFAPILVAWLFCVGGVGLYNIIHWNPGVIRAVSPYYAYKFFKVTGKVGWHSLGGIVLCITGAEAMFADLGHFSQLSLRIAFAGFVYPCLVLAYMGEAAYLSEH---REDLQSSFYKAIPEPIFWPIFVIATLATVVASQAIISATFSIISQCRALKCFPRVRIVHTSDDIYGRIYIPEANWTLMVLCLAVVLGFRDTDKIGNAYGLAVISVMFVTTCLMFLIIVTVWRRNVVVAIIFVLAFGSIELFYMSACLAKIHQGGWVPLVSSFVILCLMSVWKYGTVKKLAFEHQNKVSLERFFTL--GPSLGVLRVPGIGLIYSNVTSGVPPMFAHFVTNFPAFHQVLIFVTIETLLVPTVPVNERLEIGGVGPQELRILRCTVRYGYKDIR-DTYDFESKLIEEVVEFLQRK-----AND-------GEAPTPSPPLNHM---T---------------NGDRVSEGGGIALGRRTRRV-RFRD-------------PEPNGDVEKLMEARKAG-VAYMIGNTCVVAREESSYIKKIAINLIYRFLRQNCRRPAVALGIPHTSLIEVGMVYYI

#XP_010669975_Beta_vulgaris_subsp._vulgaris

GTFALYSLLCRFSKMGLFYPSQMGHAHPSFYESGTSKKETKCSL---AIKEFFEKHRYSRVVLLLVVLLGTSMVIGDGILTPTMSVLSAVSGLKIKIP-HLNEYCTVSVACVILLVIFSLQKYGTSRVGFLFAPILVAWLFCVGGVGLYNIIHWNPGVIRAVSPYYAYKFFKVTGKVGWHSLGGIVLCITGAEAMFADLGHFSQLSLRIAFAGFVYPCLVLAYMGEAAYLSEH---REDLQSSFYKAIPEPIFWPIFVIATLATVVASQAIISATFSIISQCRALKCFPRVRIVHTSDDIYGRIYIPEANWTLMVLCLAVVLGFRDTDKIGNAYGLAVISVMFVTTCLMFLIIVTVWRRNVVVAIIFVLAFGSIELFYMSACLAKIHQGGWVPLVSSFVILCLMSVWKYGTVKKLAFEHQNKVSLERFFTL--GPSLGVLRVPGIGLIYSNVTSGVPPMFAHFVTNFPAFHQVLIFVTIETLLVPTVPVNERLEIGGVGPQELRILRCTVRYGYKDIR-DTYDFESKLIEEVVEFLQRK-----AND-------GEAPTPSPPLNHM---T---------------NGDRVSEGGGIALGRRTRRV-RFRD-P------------EPNGDVEKLMEARKAG-VAYMIGNTCVVAREESSYIKKIAINLIYRFLRQNCRRPAVALGIPHTSLIEVGMVYYI

#NP_001390176_Oryza_sativa

GTFALYSLLCRHAKFSLLPNQQSADEELSTYYQPGVGGIISSPL-----KRFLEKHRKLRTCLLLFVLFGACMVIGDGVFTPAISVLSAISGLKDPGPGGIPDGWVVFIACIVLVGLFALQHRGTHRVAFMFAPIVVVWLLSIGVIGLYNIIHWNHRIFLALSPHYVIKFFKMTGKDGWLSLGGVLLAITGTEAMFADLGHFTAASIRLAFVGAIYPCLVLQYMGQAAFLSRN---MSAVEDSFYQSVPRSLFWPVFVIATLAAVVGSQSIISATFSIVKQCLSLGCFPRVKVVHTSRWIHGQIYIPEINWILMVLCLAVTLGFRDTTVIGNAYGLACIVVMFVTTWLMALVIIFVWQKNILLALLFVVAFGSIEVVYLSAAVTKVPQGGWAPIVFAFVFMLVMYVWHYGSRRKYLFDLQNKVSMKWILTL--GPSLGIVRVPGIGLIYTELVTGVPSIFSHFVTNLPAFHQVLVFVCVKSVPVPFVPEDERYLIGRIGPREYRMYRCIVRYGYKDVQKDDENFENHLVMSIAKFIQMEAEEAASSGSYESSEGRMAVIHTEDTTGTGLVMRDSNNEASGTSLTRSSRSETLRSLQSIYEQESGSLSRRRR-VRFEIAEEERIDPQVRDELADLLDAKEAG-VTYIIGHSYVKARKNSNFLKTFAIDYAYSFLRKNCRGPAVALHIPHISLVEVGMIYYV

#NP_196992_Arabidopsis_thaliana

GTFALYSLLCRHARISSLPNFQLADEDLSEYKKNSGENPMRLKVPGWSLKNTLEKHKFLQNMLLVLALIGTCMVIGDGVLTPAISVFSAVSGLELSMSKQQHQYVEVPVVCAILILLFSLQHYGTHRLGFVFAPIVLAWLLCISTIGVYNIFHWNPHVYKALSPYYIYKFLKKTRKRGWMSLGGILLCITGSEAMFADLGHFTQLSIQIAFTFAVYPSLILAYMGQAAYLSKHHVLQSDYRIGFYVSVPEQIRWPVLAIAILAAVVGSQAIITGTFSIIKQCTSLGCFPKVKIVHTSSRMHGQIYIPEINWTLMLLCLAVTVGFRDTKHISNASGLAVITVMLVTTCLMSLVIVLCWRKSSLYALAFIFFFGTIEVLYFSASLIKFLEGAWVPVALSFIFLLIMYVWHYGTLKRYEFDVQNKVSINWLLTLFGSSNLGIVRVHGIGVINTELVSGIPAIFSHFITNLPAFHQVVVFLCVKSVPVPHVKPEERFLVGRVGPKEYRLYRCIARYGYRDVHKDDVEFENDLICSIAEFIRSDKPLNYSPDPENESGINERLTVVAASSSNLEGVQIY----------EDDGSDKQEPSSSSEVIMVAPSPRFKKRVRFVLPESARIDRSAEEELTELTEAREAG-MAFIMGHSYVRAKSGSSVMKKIAINFGYDFLRRNSRGPCYGLSTPHASTLEVGMVYIV

#XP_004967262_Setaria_italica

GTFALYSLMCRRSRMGLLNNIH--QGPLSAYSQKEPREELKSSL---AIKGFFEKHFSLRIVLLLFVLMGTSMVIGDGVFTPTMSVLSAVSGLRIKFP-ELHENYTVLFACFVLIGLFALQHCGTHRVGFLFAPILLAWLACIGGIGIYNIFKWNPSVIRALSPYYIYNFFRKAGRDGWSSLGGIVLCITGAEAMFADLGHFSKLSLRLGFTIVVYPCLVLAYMGEAAYLSKH---REDLQSSFYKALPDTVFWPVLIIATLATVVGSQAIISATFSIISQCRALGCFPRIKVVHTSSQVHGQIYIPEVNWVLMFLCLAVTVGFRDTEMIGNAYGLSVILVMFATTCLMFLVITTVWNRNVLLAGLFTLGFGSIELTYLSACLAKVPHGGWLPLLLSLVTLLTMSTWHYGTKKKEEYELQNKVCLDRFLSL--SSGMGLVRVPGVGFIYSSAVNGVPPMFAHFVTNFPAFHRVLIFVSIQTLTVPKVSPDERFLVGRVGPPANRLFRCVVRYGYKEGRWDHFNFENQLLMKVVEFLQLQPQEDAAE---PPSDSGELSVIPASPRTHHQLADAG--------TASSASYSGGSSCEIDAGVMSRRV-RFEE--PWSGGEEGDAAAERNGEVKTLMEERESG-VSYMIGHTCVEAHESSPAVKKFAINVVYGFLRRNSRRPAAELGVPHTSLIEVGMTYRV

#XP_006351074_Solanum_tuberosum

GTFALYSLLCRHAKFSLLSNQQAADEELSAYKYGFAGQSTSCSV----LKRFLEKHKKSRTVLLIIVLLGACMVIGDGILTPAMSVISAISGIKAAAE-HLSHGEVLVLSCLILVGLFALQHSGTHRVGFLFAPIVIIWLISIFGIGLYNVIIWNPKIVHALSPYYIIKFFRETRKHGWFSLGGVLLSVAGTEAMFADLGHFTSCSMRIAFSFLVYPCLVVQYMGQAAFLSKN---LASIPDSFYNSIPDSVYWPVFVIATLASIVASQSIITATFSIVKQLNALGCFPRVKIVHTSKHVKGQIYIPEINWILMILTLSVAVGFQDTILMGNAYGLACMTSMFITTFLTTLVMIFVWQRNIVLATCFLLFFWFIEGVYLSSAFTKVPQGGWVSLVLAFVFLAVMFVWQYGTRKKYNFDLHNKVPLKWLLGM--GPSLGIVRVPGIGLVYSELATGVPAIFSHFLTNLPAFHSVLVFLCVKSVPVPYVSPEERFLIGRICPRPYRMYRCIVRYGYKDIQRDDGDFEDLLIQSIAEFIQMEAVEPQLSSSESPSLDGRMAVISKKNVQSTSTLIVSE-DFGMRDSIQSSKSLTLQSLRSAYAEENPQIRRRR--VRFQLPENPGMDPAVKAELEDLIRAKEAG-VAYIMGHSYVKARRSSSFLKKFAIDIGYSFLRKNCRGPSVVLNIPQISLIEVGMIYHV

#XP_006353127_Solanum_tuberosum

GTFALYSLLCRHAKFSLLPNQQAADEELSAYKYGSSGQSTSCLP----LKRFLEKHKKSRTILLIVVLLGACMVIGDGVLTPAMSVISSMSGIQAATE-HLSHGGVLILSCIVLVGLFALQHSGTHRVGFLFAPIVTIWLISIFLIGLYNTIFWNPKIVSALSPYYIVKFFKETGKDGWVSLGGVLLSIAGSEAMFADLGHFTATSMRIAFPFFVYPCLVVQYMGQAAFLSKN---IDSIPNSFYNSIPDSVYWPVFVIATLSAIVGSQAVITATFSIVKQCNALGCFPRVKIVHTSKHIKGQIYVPEINWILMILTLAVAVGFQDTTLIGNAYGLACMTVMFITTFLMALVIIFVWQKSVALAIPFLLLFGLIEGVYLSSAFIKIPQGGWVSLVLSFAFLTIMFVWHYGTRKKYNFDLHNKVPLKWLLGL--GPSLGIVRVPGIGLIYSELATGIPSIFSHFVTNLPAFHNVMVFVCVKSVPVPFVPPEERFLIGRICPRPYRMYRCIVRYGYKDIQRDDGNFEDLLIQSIAEFIQMEAVEPQLSSSESPSFDGRMAVISTRSVQSGSTLLVSEEDFGISNSIQSSKSLTLQSLRSAGDDENPQMRRRR--VRFRLPENPGMDPAVRDELSDLIDAKEAG-VAYIMGHSYVKARRSASFMKKLVIDIGYSFLRKNCRGPAVALNIPHISLIEVGMIYYV

#XP_008226106_Prunus_mume

GTFALYSSLCRHSRMGLLNTVHPAHERISSYCSEIPTKDTRMSL---LIKEFFEKHKSSQIVLLLVVFLGTGMIIGDGILTPTMSVLSAVYGIRIKAP-DLHENYTVFIACIILVGLFALQHYGTHKVGFLFAPIMLIWLLCISGVGMYNIFRWNPRVIGALSPYYIYNFFRKTGRIGWSSLGGVVLCLTGTEAMFADLGHFSKLSIRIAFTGLVYPCLVLAYMGEAAYLSKN---KMDISSSFYKAIPEVMFWPVLIIATLASVVGSQAIISATFSIVSQCRALRCFPRVKIKHTSNQIHGQIYIPEVNWMLMMSCLAVVIGFRDTRMIGNAYGLAGVTVMFITTCLMFLIISTVWKKNVLLAVLFFVVFGSLELLYISACLGKVHHGGWLPLLFALVIVSLMSIWNYGTMKKDAFELENKVSLDRLLSL--GPSLGIARVPGICLLYSNVAFGLPPMFAHFVTNFPAFHHTLIFVTLKSLMVPKVPVVERFHVNRIGPPELSIFRCIVRYGYKEVR-DCYNFETQLIEKVAEFLKQE-----STI-------EDMTVRGQSPNHISAAV--------------GNEVCHDIAEQRRKEVSGGSGEQWSNTGFQSL--------GSCQEMKKLMEAREAGGVAYMMGNPYIVASGVSPFLKKFAINIVYGFLRRNCRRPAIALGVPHTSLLEVGMFYQV

#XP_011022087_Populus_euphratica

GTFALYSLLCRHSRMGLLKSSHLEDEFLTCYDSNGSTKETRTSL---VIKEFFNEHRSSRVVLLLVVLLGTSMVIGDGILTPAMSVLSAVYGIQIKAP-DLHENYTVAIACVILVGVFALQHCGTHRIGFLFSPILIAWLLCISGVGIYNIFQWNPDVFKAISPYYIYNFFKKTGTAGWSSLGGIVLCATGAEAMFADLGHFSELSVRIAFTGLVYPSLVLAYMGEAAYLSKN---RRDLQSSFYKAVPDAIFLPVFIIATLATVVGSQAIISATFSIISQCRALSCFPRVKIVHTSNNIHGQIYIPEVNWVLMILCLAVVVGFRDTATIGNAYGLAVITVMLVTTLLMFLIISTVWKKHVFLAFLFVVIFGFVELSYFNACLAKLHKGGWFPLVVSAVVLSLMSIWHYGTLKKQAFELENKVSLDCLLRL--GPCMEIERVPGVCLVYSHVTSGVPPMFAHFATNFPAFHQILIFVTIQSLIVPKVPISDRFHVSRIGPPQLPLFRCIVRYGYKDIR-DSYAFETQLIEKISEFLKRD-----LSS-------EQMVVIEQSLH--------------------------------GAKTRRSRELRFQC-------------QEASEDVNELMEAEEAGMV-YMIGHTCVISNKASCILKKFVINVVYGFLRRNSRSPAASLGIPLTALIEVGRVYRV

#XP_015882215_Ziziphus_jujuba

GTFALYALLCQNSRVGLLSTGYPADKHICSCNSEISKRETRTSL---LMKEFFERYKSSRVVLLLVVLLGTSMVIGDGILTPTMSVVSAVYGIKIKAT-ELDENYTVLITCIILVGLFTLQHFGTHRVGFLFAPIMVSWLLCVGGVGIYNIFHWNPSVIRGLSPHYIYKFFRKTGRVGWNSLGGIVLCLTGAEAMFADLGHFSQLSVRIAFTGLVYPCLVLGYMGEAAYLSKN---KMDLQNSFFKAIPEPIFWPVFVIATLATMVGSQAIISATFSIISQCRALRCFPRVKIKHTSNQIHGQIYIPEANWILMLLCLAVVTGFRDTNMIGNAYGLAVITVMFITTCLMFLVICMVWKRNILLAVLFVAIFGSVEMLYISSCFAKVHKGGWLPLIISLVVMSFMSSWHYGTSKKDAFELQNKVCLDSFLSL--GPSLGIARVPGICLVYSNLTSSIPPMFAHFVTNFPAFHRILIFVNLKLFMVPNVPGGERFLVSRIGTPDLRIFRCIVRYGYKDMM-DSYNFENNLIEKVAEFLKQE-----CSS-------DNMTVRKQSPDNT---N----------------DATGDEGVDGGGAERRREVGFLSL--------------GSSEEVKELMEAKEAG-VAYMMGNTYVMASETSSFMKKIAIDVVYGFLRRNCRRPATGLGIPSTSLIEVRMLYRI

#XP_021764090_Chenopodium_quinoa

GTFALYSLLCRSSKMGFFCPSQVKYAHPS-YEAAASGKESMSSL---ALKEFFEKHYYSRLILFLVVLLGTSMVIGDGILTPTMSVLSAVSGLKVKIP-HLNEYLTLGVACIILMGIFSLQHFGTSKVGFLFAPILVAWLLCVGGVGLYNIIHWNPGVIRAVSPYYVYKFFKVTGKVGWSSLGGVVLCITGAEAMFADLGHFSQLSLRIAFSGFVYPCLVLAYMGEAAYLSKH---SEDLQSSFYKAIPESIFWPFFVIATLATVVASQAIISATFSIISQCRALKCFPRVRMVHTSDDIYGRIYIPEANWILMVLCLAVVLGFRDTDKIGNAYGLAVITVMFVTTCLMFLIIVTVWKKHVLVALFFVLFFGSIELSYMSACLIKIHQGGWVPLVSSLIILCLMSLWKYGTMKKQAFEHQNKVGLDRFLTL--GPSLGVLRVPGIGLIYSNVTTGVPPMFSHFVANFPAFHQVLIFVTIETLLIPRVPVNERLVIGRVGPLELHIFRCTVRYGYKDTR-DTYDLESKLIDEVAEFLQHGP-----H---------ESETLTSSNN-----V----------------ADTDAAPEGGAVTVGRRRV-RFREVEPS----------GPNVNVKKLMEARQSG-VAYMIGNTCVVAREESSFFKKIAINIVYRFLRQNCRRPAVALGIPHTSLIEVGMVYHV

#XP_021764091_Chenopodium_quinoa

GTFALYSLLCRSSKMGFFCPSQVKYAHPS-YEAAASGKESMSSL---ALKEFFEKHYYSRLILFLVVLLGTSMVIGDGILTPTMSVLSAVSGLKVKIP-HLNEYLTLGVACIILMGIFSLQHFGTSKVGFLFAPILVAWLLCVGGVGLYNIIHWNPGVIRAVSPYYVYKFFKVTGKVGWSSLGGVVLCITGAEAMFADLGHFSQLSLRIAFSGFVYPCLVLAYMGEAAYLSKH---SEDLQSSFYKAIPESIFWPFFVIATLATVVASQAIISATFSIISQCRALKCFPRVRMVHTSDDIYGRIYIPEANWILMVLCLAVVLGFRDTDKIGNAYGLAVITVMFVTTCLMFLIIVTVWKKHVLVALFFVLFFGSIELSYMSACLIKIHQGGWVPLVSSLIILCLMSLWKYGTMKKQAFEHQNKVGLDRFLTL--GPSLGVLRVPGIGLIYSNVTTGVPPMFSHFVANFPAFHQVLIFVTIETLLIPRVPVNERLVIGRVGPLELHIFRCTVRYGYKDTR-DTYDLESKLIDEVAEFLQHGP-----H---------ESETLTSSNN-----V----------------ADTDAAPEGGAVTVGRRRV-RFREVEPS----------GPNVNVKKLMEARQSG-VAYMIGNTCVVAREESSFFKKIAINIVYRFLRQNCRRPAVALGIPHTSLIEVGMVYHV

#XP_021828580_Prunus_avium

GTFALYSSLCRHSRMGLLNTVHPAHERISSYCSEIPTKDTRMGL---LIKEFFEKHKSSRIVLLLVVFLGTGMIIGDGILTPTMSVLSAVYGIRIKAP-DLHENYTVFIACIILVGLFALQHYGTHKVGFLFAPIMLIWLLCISGVGMYNIFRWNPRVIGALSPYYIYYFFRKTGRIGWSSLGGVVLCLTGTEAMFADLGHFSKLSIRIAFTGLVYPCLVLAYMGEAAYISKN---KMDISSSFYKAIPEVMFWPVFIIATLASVVGSQAIISATFSIVSQCRALRCFPRVKIKHTSNQIHGQIYIPEVNWMLMMSCLAVVIGFRDTRMIGNAYGLAGVTVMFITTCLMFLIISTVWKKNVLLAVLFFVVFGSLELLYISACLGKVHHGGWLPLLFALVIMSLMSIWNYGTMKKDAFELENKVSMDRLLSL--GPSLGIARVPGICLLYSNVAFGLPPMFAHFVTNFPAFHHTLIFVTLKSLMVPKVPDVERFHVNRIGPPELSIFRCVVRYGYKEVR-DCYNFETQLIEKVAEFLKQE-----STI-------EDMTVRGQSPNHISAAV--------------GNEVCRDIAEQRRKEVSGGSGEQWSNTGFQSL--------GSHQEMKKLMEAREAGGVAYMMGNPYVVASGVSPFLKKFAINIVYGFLRRNCRRPAIALGVPHTSLLEVGMLYQV

#XP_021867037_Spinacia_oleracea

GTFALYSLLCRSSRMGIFFPSQAEHAHPS-YDGGTSRKEGKTSL---VIKDFFEKHYYSRLFLLLVVLLGTSMVIGDGILTPTMSVLSAVSGIKVKIP-HLHEYITVGVACVILMAIFSLQHCGTSKVGFLFAPILVAWLLSVGGVGLYNIVHWNPGVFRALSPYYAYKFFKVTGKVGWSSLGGIVLCVTGAEAMFADLGHFSQVSLRVAFAGFVYPCLVFAYMGEAAYLSKH---IEDLQSSFYKAIPEPIFWPFFVIATLASVVASQAIISATFSIISQCRALKCFPRVKVMHTSDEIHGRIYIPEANWMLMVLCLAVVLGFRDTDKIGNAYGLAVITVMFVTTCLMFLVIVTVWKRHVLVALIFVLVFGSIELSYMSACLMKIHRGGWLPLVSSLVILCLMLVWKYGTVKKQEFEHQNKVGLDRFLTI--GPSLGVIRVPGIGLIYSNITSGVPPMFSHFVANFPAFHKVLIFVTIETLLVPRVPVNERLVISRVSLPEHHIFRCTVRYGYKDVR-DSYDFESKLVDKVEEFLQG------G----------ETLTSSPPPNNVADSD--------------TDAVPESSGTAAAAAVGRRRV-RFRDVELQS---------GPNVNVRELMEARQAG-VAYMIGNTCVVAREEASFAKKLAINMIYRFLRQNCRRPATALGIPHTSLIEVGMVYNI

#XP_024160141_Rosa_chinensis

GTFALYSSLCRHSRMGLLNTVDPAHERVSSCRSETS-KDTRMSL---LIKEFFEKHKSSRIVLVLVVFLGIGMVIGDGILTPTMSVLSAVNGIKVEVP-DLHENYTVLIACTILVSLFALQHYGTHRVGFLFAPIMTAWLLCISGVGIYNIFRWNPGVICALSPHYIYKFFEKTGRAGWSSLGGIVLCLTGTEAMFADLGHFSKLSIRVAFSGLVYPCLVLAYMGEAAYLSKH---RMDLHRSFFKAIPEDVFWPVFIIATLASVVGSQAIISATFSIVSQCRGLRCFPRVRIEHTSNQIHGQIYIPEVNWILMVLCLAVVIGFRDTNMIGNAYGLAAVTVMFITTCLMFFIISTVWKKNVLLAFLFVVIFGSLELLYISACLSKVHKGGWLPLLFAVVILSLMSIWNYGTVKKDAFELQNKVSLDRLLSS--GLSLGIKRVPGICLVYSNVASGVPPMFAHFVTNFPAFHHTLIFVTLKSLMVPKVPVDERFVINRIGPPELLIFRCIVRYGYKDVR-DCYKFETQLIEEVAEFLKQE-----AHS-------KEMAVRGQSPNFMFAAV--------------GNEVSIASSEQWRNEIKGGSV--------------------ECQKVNELKEAREAG-VAYMMGNTHVVASDLSPFLKKFAIDIVYGFLRRNCRRPAIALQIPHTSLIEVGMLYQV

#XP_034589605_Setaria_viridis

GTFALYSLMCRRSRMGLLNNIH--QGPLSAYSQKEPREELKNSL---AIKGFFEKHFSLRIVLLLFVLMGTSMVIGDGVFTPTMSVLSAVSGLRIKFP-ELHENYTVLFACFVLIGLFALQHCGTHRVGFLFAPILLAWLACIGGIGIYNIFKWNPSVIRALSPYYIYNFFRKAGRDGWSSLGGIVLCITGAEAMFADLGHFSKLSLRLGFTIVVYPCLVLAYMGEAAYLSKH---REDLQSSFYKALPDTVFWPVLIIATLATVVGSQAIISATFSIISQCRALGCFPRIKVVHTSSQVHGQIYIPEVNWVLMFLCLAVTVGFRDTEMIGNAYGLSVILVMFATTCLMFLVITTVWNRNVLLAGLFTLGFGSIELTYLSACLAKVPHGGWLPLLLSLVTLLTMSTWHYGTKKKEEYELQNKVCLDRFLSL--SSGMGLVRVPGVGFIYSSAVNGVPPMFAHFVTNFPAFHRVLIFVSIQTLTVPKVSPDERFLVGRVGPPANRLFRCVVRYGYKEGRWDHFNFENQLLMKVVEFLQLQPQEDAAEP---PSDSGELSVIPASPRAHHQLADAG--------TASSASYSGGSSCEIDAGVMSRRV-RFEE--PWSGGEEGDAAAERNGEVKTLMEERESG-VSYMIGHTCVEAHESSPAVKRFAINVVYGFLRRNSRRPAAELGVPHTSLIEVGMTYRV

#XP_044424600_Triticum_aestivum

GTFALYSLMCRRSRMGLLNSLHAGHGSVSSYNQDEPCKETRSSL---AIRGFFEKHHSLRVVLLLFVLMGTSMVIGDGVLTPTMSVLSAVSGLRIKFP-ELHENYTVLLACVVLVGLFALQHYGTRRVGFLFAPILLSWLACIGGIGIYNIFRWNPTVVRALSPYYIYNFFRKAGRDGWSSLGGIVLCITGAEAMFADLGHFSKLSLRLGFTIVVYPCLVLAYMGEAAYLSKH---REDLQSSFYKALPDRVFWPVLFIATLATAVGSQAIISATFSIISQCRALGCFPRIKVVHTSSHVHGQIYIPEVNWTLMSLCLAVTIGFRDTEMIGNAYGLAVILVMFATTCLMFLVITTVWNRSVLWAALFAAGFGSMELLYLSACLAKVPHGGWLPLLLSLATLLVMSAWHYGTAKKQEYEMQNKVCLDHFIGL--SSGMGLVRVPGVGFVYSDSVAGVPPMFAHFVTNFPAFHRVLVFVSLQTLTVPKVPPEERFLVGRIGLPEHRMFRCVVRYGYKEGRWDHFNFEDQLLVKVLEFLQLQQADGDGERCSTGS--GEMSVIPAAPSQAVVDA---------------LASMSSGEIEYYAGGGAKKV-RFEELPAAWRRE------ETMVEVRALLEEREAG-VSYMIGHTCVFAHESSSAVKKFAVNVVYGFLRRNSRRPAVVLGIPHTSLIEVGMVYRV

#XP_044439792_Triticum_aestivum

GTFALYSLMCRRSRMGLLNSLHAGHGSVSSYNQDEPCKETRSSL---AIRGFFEKHHSLRVVLLLFVLMGTSMVIGDGVLTPTMSVLSAVSGLRIKFP-ELHENYTVLLACVVLVGLFALQHYGTRRVGFLFAPILLSWLACIGGIGIYNIFRWNPTVVRALSPYYIYNFFRKAGRDGWSSLGGIVLCITGAEAMFADLGHFSKLSLRLGFTIVVYPCLVLAYMGEAAYLSKH---REDLQSSFYKALPDRVFWPVLFIATLATAVGSQAIISATFSIISQCRALGCFPRIKVVHTSSHVHGQIYIPEVNWTLMSLCLAVTIGFRDTEMIGNAYGLAVILVMFATTCLMFLVITTVWNRSVLWAALFAAGFGSMELLYLSACLAKVPHGGWLPLLLSLATLLVMSAWHYGTAKKQEYELQNKVCLDHFIGL--SSGMGLVRVPGVGFVYSDSVAGVPPMFAHFVTNFPAFHRVLVFVSLQTLTVPKVPQEERFLVGRIGRPEHRMFRCVVRYGYKEGRWDHFNFEDQLLVKVLEFLQLQQADGDGERCSTGS--GEMSVIPAAPSQAVVDA---------------LASMSSGEIEYYAGGGAKKV-RFEELPAAWRRE------ETMSEVRELLEEREAG-VSYMIGHTCVFAHESSSAVKKFAVNVVYGFLRRNSRRPAVVLGIPHTSLIEVGMVYRV

#XP_048229046_Ricinus_communis

GTFALYSLLCRRSKMGFLLSSHMGLECVSSHDSSLPARETRTSL---IIKEFFEKHHSSRIVLLLVVLLGTSMVIGDGILTPTMSVLSAVYGIQIKLP-NLHENYTVVIACVVLVGLFALQHYGTHRVGFVFAPILLAWQLCLGGIGIYNIFHWNPGVINALSPHYIYKFFQRAGKSGWSSLGGIILCVAGAEAMFADLGHFSKLSLRIAFTVVVYPCLVLAYMGEAAYLSKH---KEDLQRSFYKAIPEAIFWPVFLIATLATVVGSQAIISATFSIISQCRALGCFPRVKIVHTSKNIHGQIYIPEVNWLLMVFCLAVVIGFRDTSMIGNAYGLAVIIVMFVTTLLMFLIISTVWKRNVSWAIIFVLVFGSVELSYLSACLAKVHKGGWLPLLVSLVISSLMSIWRYGTSKKLAYELDNKVSLDSLLSV--GASLGMTRVPGICLVYSDITSGVPPMFAHFITNFPAFHEILIFVTLQSLMIPKVPIDERFHIVRIGPPEFSLFRCIVRYGYKDIK-DSHALETQLIEIISGFLKSERQ---GK---------EIAVMDTIRK-------------------------------GGRPTDGRKKVSFQLHNL-----------EANEEIKGLMEAKEAG-VAYMMSNTSVRANEASSFVKKFAINIVYAFLRRNSRCPATALGIPHPSLIEVGMVYLV

#XP_050107045_Malus_sylvestris

GTFALYSSLCRHSRLGLWNTVHPEYESIPSDCSGLSTKDTRMSS---LIKNFFEKHKSSKIVLLLVVFIGVGMIIGDGILTPTMSVLSAVSGIRIKAP-DLHENYTVLIACIILVGLFALQHFGTHKVGFLFAPIMLTWLLCICGVGIYNIFRWNPGVICALSPYYIYNFFKITRRVGWSSLGGVVLCVTGTEAMFADLGHFSKLSIRIAFTALVYPCLILAYMGEAAYLSKH---KKDLRCSFYMAIPEVMFWPVVIIATLASAVGSQAIISATFSIVSQCRALRCFPRVKIKHTSNQIHGRIYIPEVNWMLMVLCLAVVIGFRDTHMIGNAYGLAGVTVMFITTCLMFLIISTVWKQKVIMAFLFFVIFGSLELLYISACLGKAHHGGWLPLLFALVIVSLMSIWNYGTVKKDAFELDNKVSLDRLLSF--GPSLGITRVPGICLVYSKVAFGLPPMFAHFVTNFPTFHHTLIFVTLKYLMIPKVPLGERFLVNRIGPPELSIFRCIVRYGYKDVK-DCYNFETQLIEKVAEFLKQE-----RNS-------EELAVRGQSPNQISAAT--------------RNEVYGGRAVHWSDDVSGGSSEQWRDGVG-----------SAKQWMKKLMEAREAGGVTYMMGNPYVEASEVSPFLKKFAIDIVYNFLRRNCRRPAVTLGIPHTSVIEVGMLYQV

#XP_056693632_Spinacia_oleracea

--------------MGIFFPSQAEHAHPS-YDGGTSRKEGKTSL---VIKDFFEKHYYSRLFLLLVVLLGTSMVIGDGILTPTMSVLSAVSGIKVKIP-HLHEYITVGVACVILMAIFSLQHCGTSKVGFLFAPILVAWLLSVGGVGLYNIVHWNPGVFRALSPYYAYKFFKVTGKVGWSSLGGIVLCVTGAEAMFADLGHFSQVSLRVAFAGFVYPCLVFAYMGEAAYLSKH---IEDLQSSFYKAIPEPIFWPFFVIATLASVVASQAIISATFSIISQCRALKCFPRVKVMHTSDEIHGRIYIPEANWMLMVLCLAVVLGFRDTDKIGNAYGLAVITVMFVTTCLMFLVIVTVWKRHVLVALIFVLVFGSIELSYMSACLMKIHRGGWLPLVSSLVILCLMLVWKYGTVKKQEFEHQNKVGLDRFLTI--GPSLGVIRVPGIGLIYSNITSGVPPMFSHFVANFPAFHKVLIFVTIETLLVPRVPVNERLVISRVSLPEHHIFRCTVRYGYKDVR-DSYDFESKLVDKVEEFLQG------G----------ETLTSSPPPNNVADSD--------------TDAVPESSGTAAAAAVGRRRV-RFRDVELQS---------GPNVNVRELMEARQAG-VAYMIGNTCVVAREEASFAKKLAINMIYRFLRQNCRRPATALGIPHTSLIEVGMVYNI

#XP_057987636_Hevea_brasiliensis

GTFALYSLLCRRSKMGFLKSSNTGHGCLSSHESCVPTKETRTSL---LIKEFFEKHHSSRVVLLLVVLLGTSMVIGDGILTPTMSVLSAVYGIQIKVP-HLHENYTMAIACVVLVGLFALQHYGTHRVGFLFAPILLAWLLCLGGVGIYNIFHWNPGVINSLSPYYIYKFFQKTGKSGWSSLGGIVLCVTGTEAMFADLGHFSQLSLRVAFTVIVYPCLVLAYMGEAAYLSKH---KDDLQRSFYKAIPEVIFWPVFLIATLATVVGSQAIISATFSIISQSRALGCFPRVKIVHTSNNIHGQIYIPEVNWMLMLLCLAFVIGFRDTAMIGNAYGLAVVVVMFATTLLMFLIISSVWNQNVLWAFLFVLVFGFVELSYFSACLAKVHKGGWLPLVVSLLILSLMSTWHYGTSKKLAFELENKVSLDSLLTL--GSNLGIVRVPGICLVYSDVTNGAPPMFAHFVTNFPAFHQILIFVTLQSLMIPKVPVGDRFHIARMGPPEFSLFQCIVRYGYKDVR-DSHDLETHMIENMSRFLKCE-----GHS-------KEMAMTEPTQN---------------------------------GGTGTRKL-RFQITM------------EASQEVGELMEAKEAG-VAYIIGNTIVRASGASCFLKKFAIDIVYGFLKRNSRSPATALGIPTTSLVEVSIVYRV

#XP_057988859_Hevea_brasiliensis

GTFALYSLLCRRSNLGFLKSSNMGHGCLSSHESCVPTKETRTSL---LIKEFFEKHHSSRVVLLLVVLLGTSMVIGDGILTPTMSVLSAVYGIQIKVP-HLHENYTMAIACVVLVGLFALQHYGTHRVGFLFAPILLAWLLCLGGVGIYNIFHWNPGVINSLSPYHIYKFFQKTGKSGWSSLGGIVLCVTGTEAMFADLGHFSQLSLRVAFTVIVYPCLVLAYMGEAAYLSKH---KDDLQRSFYKAIPEVIFWPVFLIATLATVVGSQAIISATFSIISQSRALGCFPRVKIVHTSNNIHGQIYIPEVNWMLMLLCLAFVIGFRDTAMIGNAYGLAVVVVMFATTLLMFLIISTVWNQNVLWAFLFVLVFGFVELSYFSACLAKVHKGGWLPLVVSLLILSLMSTWRYGTSKKLAFELENKVSLDSLLTL--GSNLGIVRVPGICLVYSDVTNGAPPMFAHFVTNFPAFHQILIFVTLQSLMIPKVPVGDRFHIARIGPPEFSLFQCIVRYGYKDVK-DSHDLETHMIENMSRFLKCE-----GHS-------EEMAMTEPTQN---------------------------------GGTGTRKL-RFQINM------------EASQEVGELMEAKEAG-VAYIIGNTIVRASGASCFLKKFAIDIVYGFLKRNSRSPATALGIPTTSLVEVSIVYRV

#XP_062012005_Rosa_rugosa

GTFALYSSLCRHSRMGLLNTVDPAHERLSSCRSETS-KDTRMSL---LIKEFFEKHKSSRIVLVLVVFLGTGMVIGDGILTPTMSVLSAVNGIKVKVP-HLHENYTVLIACTILVSLFALQHYGTHRVGFLFAPIMTAWLLCISGVGIYNIFRWNPGVICALSPHYIYKFFEKTGRAGWSSLGGIVLCLTGTEAMFADLGHFSKLSIRVAFSGLVYPCLVLAYMGEAAYLSKH---RMDLHRSFFKAIPEDVFWPVLIIATLASVVGSQAIISATFSIVSQCRGLRCFPRVKIKHTSNQIHGQIYIPEVNWILMVLCLAVVIGFRDTNMIGNAYGLAAVTVMFITTCLMFLIISTVWKKNVLLAFLFVVIFGSLELLYISACLSKVHKGGWLPLLFAVVILSLMSIWNYGTVKKDAFELQNKVSLDRLLSS--GPSLGITRVPGICLVYSNVASGVPPMFAHFVTNFPAFHHTLIFVTLKSLMVPKVPVDERFVVNRVGPPELLIFRCIVRYGYKDVR-DCYEFETQLIEEVAEFLKQE-----AHS-------KEMAVREKSPNCMFAAV--------------GNEVSIASSEQLRNKIKGGSV--------------------ECQEVNELKEAREAG-VAYMMGNTHVVASDLSPFLKKFAIDIVYGFLRRNCRRPAIALQIPHTSLIEVGMLYQV

#XP_062234004_Phragmites_australis

GTFALYSLMCRRSRMGLLNNIHADHGSLPAYNQEELREEPKSSL---AIKGYFEKHYSLRVVLLLFVLMGTSMVIGDGVFTPTMSVLSAVSGLRIKFP-ELHENYTVLIACVVLVGLFALQHYGTHRVGFLFAPILLAWLCCIGGIGIYNIFKWNLSVVRALSPYYIYNFFRKTGKDGWSSLGGIVLCITGAEAMFADLGHFSKLSLRLGFTIVVYPCLVLAYMGEAAYLSKH---REDLQSSFYKALPDSVFWPVLIIATLATVVGSQAIISAAFSIISQCRALGCFPRIKVVHTSSHVHGQIYIPEVNWVLMLLCLAVTAGFRDTEMIGNAYGLAVILVMFATTCLMFLVITTVWNRNVLWAVLFTFGFGSIELMYLSACLAKVPHGGWLPLLLSLVTLLAMSAWHYGTKKKEEFELQNKVCLDRFLSL--SSGIGLVRVPGVGFVYSSAVNGVPPMFAHFVTNFPAFHRVLIFVSLQTLTVPKVSPNERFLVGRVGPPANRLFRCVVRYGYKEGRWDHFNFENELLMKVVEFLQLQEDAAELDDS------GELSVIPAAPHSQLLVDVDA----------APMASYSSSSGEIDAGVVARRV-RFEEPCTGE--------ETKSSEVKTLLEERESG-VSYMIGHTCVQAHESSSAVKKFAVNVVYGFLRRNSRRPAVELGIPHTSLVEVGMTYRV
