# Supplementary figures and images for: Overexpression of BvKUP13 from sugar beet increased salt tolerance in transgenic Arabidopsis thaliana
Source: Front Plant Sci. 2026 Feb 23;17:1736699. doi: 10.3389/fpls.2026.1736699 (PMC12970625; doi:10.3389/fpls.2026.1736699)

M OE3 OE5 OE9 WT ddH2O Plasmid

1 2 3 4 5 6


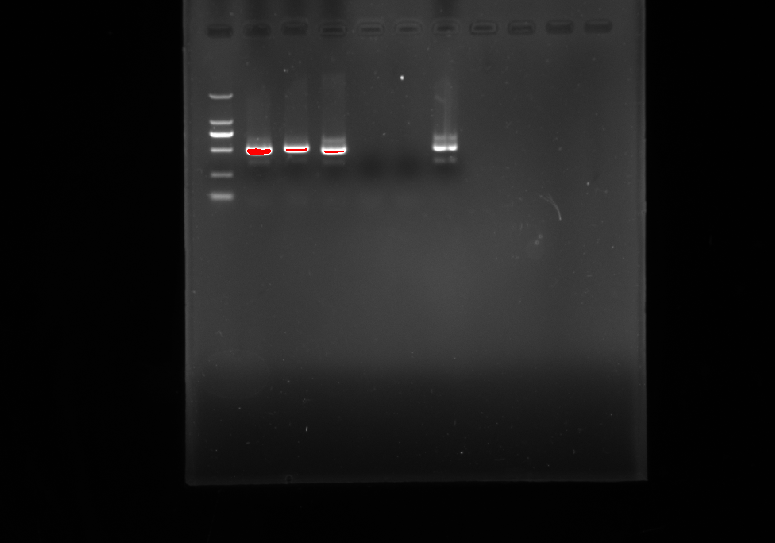

Supplement: Supplementary file 1 [file DataSheet1.zip › Supplement-Wang-Experimental Data/fig4.4B.docx]
