# Supplementary material for: Overexpression of BvKUP13 from sugar beet increased salt tolerance in transgenic Arabidopsis thaliana
Source: Front Plant Sci. 2026 Feb 23;17:1736699. doi: 10.3389/fpls.2026.1736699 (PMC12970625; doi:10.3389/fpls.2026.1736699)
Supplement: Supplementary file 2 [file DataSheet2.pdf]

Table S1 Primers used for experimentation

Primers	Forward(5'-3')	Reverse(5'-3')
BvKUP13	-ATGGATTTATCAACTCACCC	-GTCATATATAGTAAACCATTCCTAC
gfpBvKUP13	-AAGTCCGGAGCTAGCTCTAG atggatttatcaactcacccatctcgatttgag	-AGCGGCCGCTGTACAGGATCtatatagt aaaccattcctacttcaatcagtgatgtatgc
BvActin	-TGCTTGACTCTGGTGATGGT	-AGCAAGATCCAAACGGAGAATG
qBvKUP13	-AAGGGCACTGTGTCTTGCAT	-CTTCGTAAAGCCGCAAGCTC
Kan	-TTGTCACTGAAGCGGGAAG G	-CGGCGATACCGTAAAGCAC
AtActin2	CTGGATTCTGGTGATGGTGTG TCT	GAACCACCGATCCAGACACTGTAC
pBI101-BvKUP 13-F/R	GAGAACACGGGGGACTCTAG AACATGATGGATTTATCAACT CACCCATCTC	CTTGGACGTTGCAAACGTAAGTGTT GTCATATATAGTAAACCATTCCTAC
GFPBvKUP1 3-R/F	CCTATAGCCTACATTATACGatg gatttatcaactcacccatctcgatttgag	TCCTCGCCCTTGCTCACCATagatcctcc tccagatcctcctc
